# Automatic Choroid Layer Segmentation from Optical Coherence Tomography Images Using Deep Learning

**DOI:** 10.1038/s41598-019-39795-x

**Published:** 2019-02-28

**Authors:** Saleha Masood, Ruogu Fang, Ping Li, Huating Li, Bin Sheng, Akash Mathavan, Xiangning Wang, Po Yang, Qiang Wu, Jing Qin, Weiping Jia

**Affiliations:** 1 Department of Computer Science and Engineering, Shanghai Jiao Tong University, Shanghai, 200240 China; 2 J. Crayton Pruitt Family Department of Biomedical Engineering, University of Florida, Gainesville, FL 32611 USA; 3 Faculty of Information Technology, Macau University of Science and Technology, Macau, 999078 China; 4 Shanghai Jiao Tong University Affiliated Sixth People’s Hospital, Shanghai, 200233 China; 5 Department of Computer Science, Liverpool John Moores University, Liverpool, L3 3AF UK; 6 Centre for Smart Health, School of Nursing, The Hong Kong Polytechnic University, Hong Kong, 999077 China

**Keywords:** Endocrinology, Health care

## Abstract

The choroid layer is a vascular layer in human retina and its main function is to provide oxygen and support to the retina. Various studies have shown that the thickness of the choroid layer is correlated with the diagnosis of several ophthalmic diseases. For example, diabetic macular edema (DME) is a leading cause of vision loss in patients with diabetes. Despite contemporary advances, automatic segmentation of the choroid layer remains a challenging task due to low contrast, inhomogeneous intensity, inconsistent texture and ambiguous boundaries between the choroid and sclera in Optical Coherence Tomography (OCT) images. The majority of currently implemented methods manually or semi-automatically segment out the region of interest. While many fully automatic methods exist in the context of choroid layer segmentation, more effective and accurate automatic methods are required in order to employ these methods in the clinical sector. This paper proposed and implemented an automatic method for choroid layer segmentation in OCT images using deep learning and a series of morphological operations. The aim of this research was to segment out Bruch’s Membrane (BM) and choroid layer to calculate the thickness map. BM was segmented using a series of morphological operations, whereas the choroid layer was segmented using a deep learning approach as more image statistics were required to segment accurately. Several evaluation metrics were used to test and compare the proposed method against other existing methodologies. Experimental results showed that the proposed method greatly reduced the error rate when compared with the other state-of-the-art methods.

## Introduction

The choroid layer is vital for the oxygenation and metabolic activity of the Retinal Pigment Epithelium (RPE) and outer retina. It is a vascular interface between the retina and sclera and requires some of the highest amounts of blood flow for any tissue in the human body. The choroid layer also acts as a source of blood supply for the optic nerve^1^. The structure of the layer is mainly divided into two parts based on anatomical structure: the vascular plexus, consisting of various capillaries contiguous to BM, and the choroidal stroma. Changes in the shape and anatomical structure of the choroid have been acknowledged in primary macular degeneration and in other advanced diseases. Quantitative and qualitative analysis of BM and the choroid layer can help in understanding the relationship between these various retinal diseases.

Optical coherence tomography (OCT) is an increasingly significant modality for the identification, monitoring, and measurement of many retinal and macular diseases as it aids in resolving cross-sectional details of the human retina. The growing need for OCT in retinal disease analysis necessitates investigation into a fully automated approach^2^. Useful information can be extracted with the blend of OCT, image processing, and segmentation techniques. This can provide detailed information regarding different retinal layers and the associated diseases. DME, a chief source of vision damage in patients suffering from diabetes, can be diagnosed with the help of choroid thickness maps as changes in thickness, such as those resulting from choroidal macular degeneration and other advanced diseases, can provide a relative measure of health of the choroid layer. Aging may also cause choroidal thinning that leads to a condition referred to as choroidal atrophy^3,4^. Increased choroidal thickness can result in diseases like serous retinopathy, polypoidal choroidal vasculopathy, autoimmune diseases, etc. Early diagnosis of these diseases can prove to be very beneficial in the treatment of these abnormalities. Thus, quantification of pathological changes can be effectively achieved by the analysis of retinal thickness and, for such diagnoses, choroidal thickness measurement is essential^5,6^.

**Figure 1:**
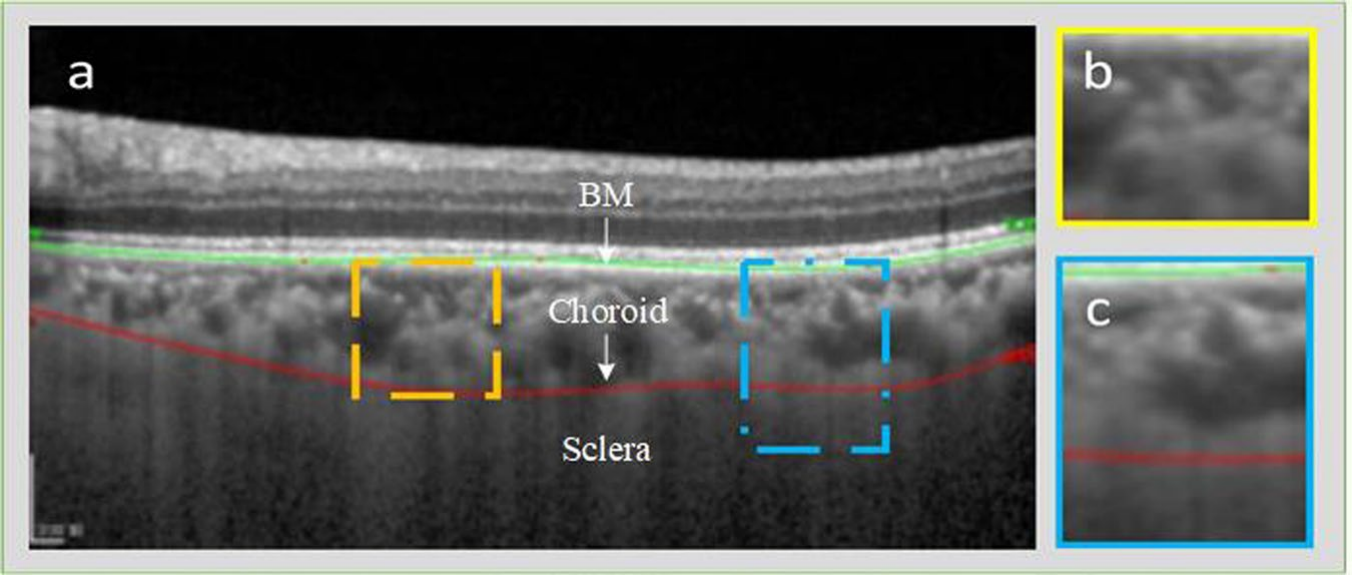
The challenges being faced by the segmentation of choroid layer: (**a**) represents OCT image B scan with ground truth being marked by the specialist. (**b**) Shows the choroidal region inhomogeneous texture. (**c**) Illustrates the inseparable interface among the sclera and choroid layer.

In OCT imaging, some approaches find the correlation among the measurable and morphological topographies of retinal thickness maps^7^. Such normal standards for the thickness maps can help physicians in comparing different patients’ choroidal thickness maps with normal sets. Consequently, the automatic segmentation of the choroidal boundary has garnered the attention of many researchers worldwide. A review of existing approaches in this domain shows that not much work has been carried out for automatic segmentation of the choroid layer, as most methods only manually or semi-automatically segment out the region of interest. Manually outlining the choroid boundary is a tedious, time consuming and sometimes impossible task because of possible indistinct structures and boundaries. Moreover, the measurement lacks objectivity, requires the trainer to be trained perfectly, and is vulnerable to inter-observer imprecision^8,9^. Also, while some fully automatic segmentation methods do exist, there is still room for improvement. Major challenges in the accurate segmentation of the choroid layer are visualized in Fig. 1 and are detailed as follows:

- Low contrast of OCT images makes the region between the sclera and choroid inseparable, resulting in a likewise inseparable histogram between the two layers
- Methods based on thresholding and intensity are not effective because of the aforementioned low contrast in OCT images
- Due to the presence of the vascular structure, the choroid layer has inconsistent texture and inhomogeneous intensity that makes extraction of the region of interest difficult
- The anatomical structure of BM and the choroid layer interface is quite weak and is often invisible

In consideration of current challenges within the choroid segmentation domain, the major contributions of this work include:

- A two-stage segmentation approach with emphasis on segmentation accuracy and consistency of the method
- OCT image segmentation based on the combination of morphological and deep learning methodologies
- Testing on the data of real patients, with experimental outcomes showing that the technique achieved high precision and reduced error rates to a significant extent

The remainder of the paper is structured as follows: Section 2 outlines literature review. Proposed methodology details are described in Section 3. Experimental results with a quantifiable examination are presented in Section 4. Finally, the conclusion and future directions are discussed in Section 5.

## Related Work

Existing literature in the context of choroid layer segmentation includes manual, semi-automatic and few automatic segmentation methods in OCT images. As the prime objective of this research focuses on the use of deep learning methodologies, the literature review is divided into two subgroups: deep learning methods and non-deep learning methods.

Machine learning is a method of data analysis that employs statistical and analytical tools on training data. These tools are later used to learn from past examples and employ the learned information from the preceding training to categorize new data, envisage new inclinations and find new patterns. Image classification or segmentation is one of the fundamental methods in the domain of machine learning. Analysis of literature shows the use of several, non-deep learning, traditional methods such as graph-based, k-nearest neighbor, Bayesian network, support vector machine and decision tree approaches, all of which involve the use of hand-crafted features for the specific classification purpose. The hand-crafted features include shape, pixel density and texture of the image features.

In the context of these non-deep learning methods, a previous automatic choroid layer segmentation method was used to extract choroidal vessels for quantification of choroidal vasculature thickness^10^. This approach focused on the thickness of vessels rather than the choroid layer. Another automatic segmentation technique^11^ applied a statistical model on choroid layers in OCT images. However, the processing time of the method is quite high and extensive training is required. Use of phase information for automatic segmentation of the interface between the choroid and sclera was proposed and implemented^12,13^. While successful, these methods are not clinically practical as the used imaging modalities are not commercially obtainable. The use of dynamic programming (DP) in one study was used to find the shortest path of a graph for choroid layer segmentation, giving a segmentation accuracy of about 90 percent^8^. Additionally, a two-stage active contour model for choroidal boundary extraction with a segmentation accuracy of 92.7 percent was proposed^14^. Graph based approaches have also been used in this context but, due to the heterogeneous nature of OCT images, these methods are not generally helpful in choroid layer segmentation^15–17^. Still, one study utilized a graph search algorithm from 3D OCT volumes to perform choroid layer segmentation^18^. This method was a semi-automatic approach. The interface between the choroid and sclera using Dijkstra’s algorithm was investigated^9^.

OCT B-scan choroidal segmentation based on a dual probability gradient has also been attempted^19^. Another study presented an automatic segmentation method based on a multi-resolution textural graph cut method^16^. The combination of Markov Random Field (MRF) and level set approach was also used to segment the choroid layer^20^. Distance regularization and edge constraint terms were rooted into the level set technique to evade uneven and trivial areas and preserve information around the boundary between the choroid and sclera. MRF based methods^21–23^ have been proposed and implemented to detect the intra-retinal layers from 2D or 3D OCT images. Another method focused on obtaining the spatial distribution of choroidal sub-layers with 3D 1060-nm OCT mapping using Haller’s and Sattler’s layer^24^. A hybrid approach has made the use of level set, multi-region continuous max-flow method to segment out different retinal layers. The approach also used nonlinear anisotropic diffusion in order to eliminate the spackle noise present in the OCT images^25^. Seven layers of the retina were also segmented in this context; the proposed approach involved a combination of graph cut and dynamic programming^26^. Two sequential diffusion map based segmentations of intra-retinal layers from 3D SD-OCT scans have been developed^27^. A similar approach for the segmentation of multiple retinal layers utilized spectral rounding for segmentation^28^.

### Review of Recent Deep Learning Techniques in Computer Vision

The conducted literature review showed that most of the existing choroid layer segmentation approaches made use of non-deep learning methodologies. An overview of existing deep learning methods revealed that, in this context, these methods are not used specifically for the choroid layer segmentation. They are generally applied in medical image segmentation, such as on the brain, retina, liver, knee, urinary bladder, chest, heart, etc. Deep learning is a branch of Artificial Intelligence (AI) with the ability to make use of optimization, probabilistic and statistical tools; these methods maintain an extensive contribution to the analysis of medical images.

With regard to biomedical image segmentation, one major study contributed an approach for the segmentation of biomedical image segmentation using Convolutional Neural networks (CNNs)^29^. The model employed a network and training strategy that relied on the robust use of data augmentation. Low grade glioma assessment through a modified CNN architecture with 6 convolutional layers and a fully connected layer was used to classify these brain tumors^30^. A similar approach was used in the diagnosis of white matter hyperintensities from brain MRI images through a series of CNN architectures that considered multi-scale patches to yield the obvious position of required features while training^31^. Another group proposed an automatic computer-aided diagnosis method for the classification of solid and non-solid nodules in pulmonary computerized tomography (CT) images through a CNN^32^. Brain image segmentation to produce high nonlinear mappings between inputs and outputs using deep learning was analyzed; the segmentation problem was solved using CNNs^33,34^. The approach made use of local features in conjunction with more global contextual features to perform the segmentation. Other studies have investigated the use of automatic segmentation and assessment of rectal cancers from the multi-parametric MR images through the use of CNN architectures^35^. The combination of CNN and total kidney volume calculation from Computed Tomography for the segmentation has been proposed in^36^. The method was tested on the images of real patients demonstrating trivial to adequate function to severe renal inadequacy. The detection of bladder cancer has also incorporated use of CNN architectures in a combination with level set methods to acquire the region of interest^37^.

In the domain of retinal image segmentation using deep learning approaches, segmentation of the optic disc, fovea and retinal vasculature has been carried out using a CNN model. The approach segmented three channels of input from the point’s neighborhood and propagated the response across the 7-layer network. The output layer was comprised of four neurons, denoting background, blood vessels, optic disc, and fovea^38^. Another study showed segmentation of retinal blood vessels using a deep neural network with zero phase whitening, global contrast normalization, and gamma corrections^39^. A similar approach for blood vessel segmentation using deep learning has been performed^40^. Segmentation of retinal blood vessels has also been analyzed as a multi-label implication task through the use of implied benefits of the blend of convolutional neural networks and the structured estimate^41^. The main observation in the overview of retinal based segmentation using deep learning is that the imaging modality being used in these approaches is the fundus image. The use of OCT images for the segmentation of retinal layers has not observed in existing methods of deep learning. As OCT imaging technology allows capturing of the cross-section of the retina, it may be very helpful to analyze OCT images for the diagnosis of several retinal diseases. Because choroid layer segmentation and associated thickness measurements help diagnose retinal based diseases, several approaches have been proposed and implemented to diagnose them. For instance, retinitis pigmentosa^42^, central serous chorioretinopathy^43^, age-related macular degeneration^44^, and diabetic retinopathy^45,46^ have been found to have associated changes in the thickness of the choroid.

One study showed the development of an automatic method for the segmentation of retinal layers based on deep learning methodologies, highlighting one of only few current implementations of an automatic technique. The method made use of CNN to perform the retinal layer segmentation in OCT images^47^. The procedure was limited in accuracy of edge detection as it depended on at most one-pixel accuracy; however, the Bidirectional Long Short-term Memory (BLSTM) entails sub-pixel accuracy. This can lead to confusion in the accurate segmentation of the corresponding boundaries. A similar method^48^ performed OCT image semantic segmentation through fully convolutional neural networks, but the results were tested on a data-set of normal individuals with fewer images of mild spectrum diabetic retinopathy. A combination of CNNs and graph search methods has also been employed in the segmentation of 9 retinal boundaries^49^. The method was computationally expensive and the black-boxed architecture of the CNN made customization and performance examination of every step less controllable. Segmentation of retinal layers with emphasis on fluid masses has also been performed using deep learning methods^50^. While results were promising, evaluation was conducted on a limited number of B-scans. The tested and training data sets contained a total of 110 B-scans.

According to analysis of the related works, it can be observed that higher segmentation accuracy was achieved through deep learning methodologies. The key restraint of non-deep leaning approaches is that these methods mainly rely on the feature extraction phase for the accurate segmentation of the region of interest. It is tough to extract appropriate image features for a definite medical image recognition problem. As a result, the classifier cannot provide effective segmentation accuracy because the segmented features are not effective enough. In order to tackle problems faced by non-deep learning approaches, significant segmentation accuracy is achieved by the use of deep learning methods through adaptive learning of image features. Considering this, it is likely that the application of deep learning methods on the categorization of OCT images for automated disease diagnosis would be more successful than counterpart non-deep learning procedures. Thus, the focus of this research is to overcome existing challenges in choroid layer segmentation and provide a fully realized automatic segmentation approach. The proposed method makes use of deep learning to achieve the desired task, with a combination of morphological operations and CNNs. In order to calculate the thickness map of choroid layer, segmentation was considered for two layers. The desired layers included BM and the choroid layer. BM was segmented out using a series of morphological operations followed by the use of CNNs for choroid segmentation. Then, a thickness map was generated based on the extracted layers.

## Material and Methods

### Pipeline Overview

**Figure 2:**
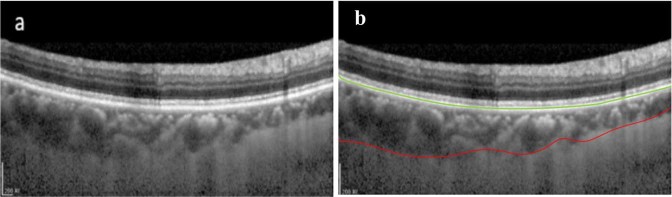
(**a**) Represents a Raw B-scan of the OCT image. (**b**) Contains OCT image manually segmented by the experts, the image contains segmented BM and Choroid layer where BM is marked in green and choroid layer is marked in red.

The proposed methodology is a two-stage segmentation process. Quantification of choroidal thickness relies on the extraction of two layers from OCT images. The segmentation of BM was carried out followed by the extraction of choroid layer from the OCT images. Figure 2 represents a standard OCT image with the required layers segmented in different colors. The regions of interest are labeled by the experts manually; BM is labeled in green whereas the choroid layer is labeled in red.

**Figure 3:**
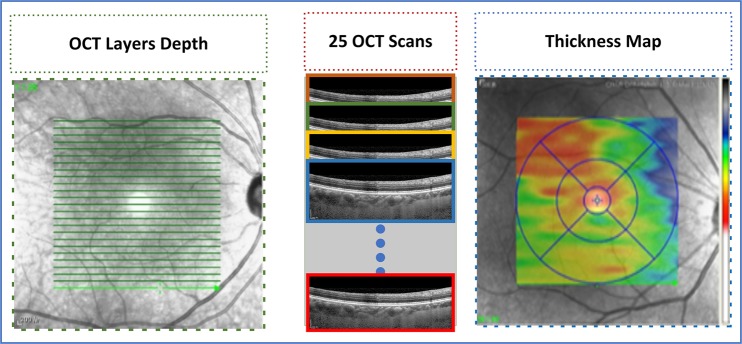
Thickness Maps: 25 b-scans of every individual were taken into account to generate a thickness map of an individual.

The proposed method takes a set of OCT images focused on the retina around the macula as input and depicts the position of two required boundaries. As the data of every individual contained 25 B-scans of the macula, the OCT images were comprised of a series of B-scans that the proposed method segmented individually. The result of segmentation of all B-scans was examined, combined and, lastly, incorporated to shape graphical depictions as 2D maps. Initially, we regulated tomography volume data by representing it as a sorted series of adjacent B-scans. Later the images were labeled by image statistics from adjacent B-scans and the region of interest was segmented in every B-scan individually. Then, the 2D retinal and choroidal thickness maps were generated. Figure 3 depicts the procedure carried out to generate the thickness map.

**Figure 4:**
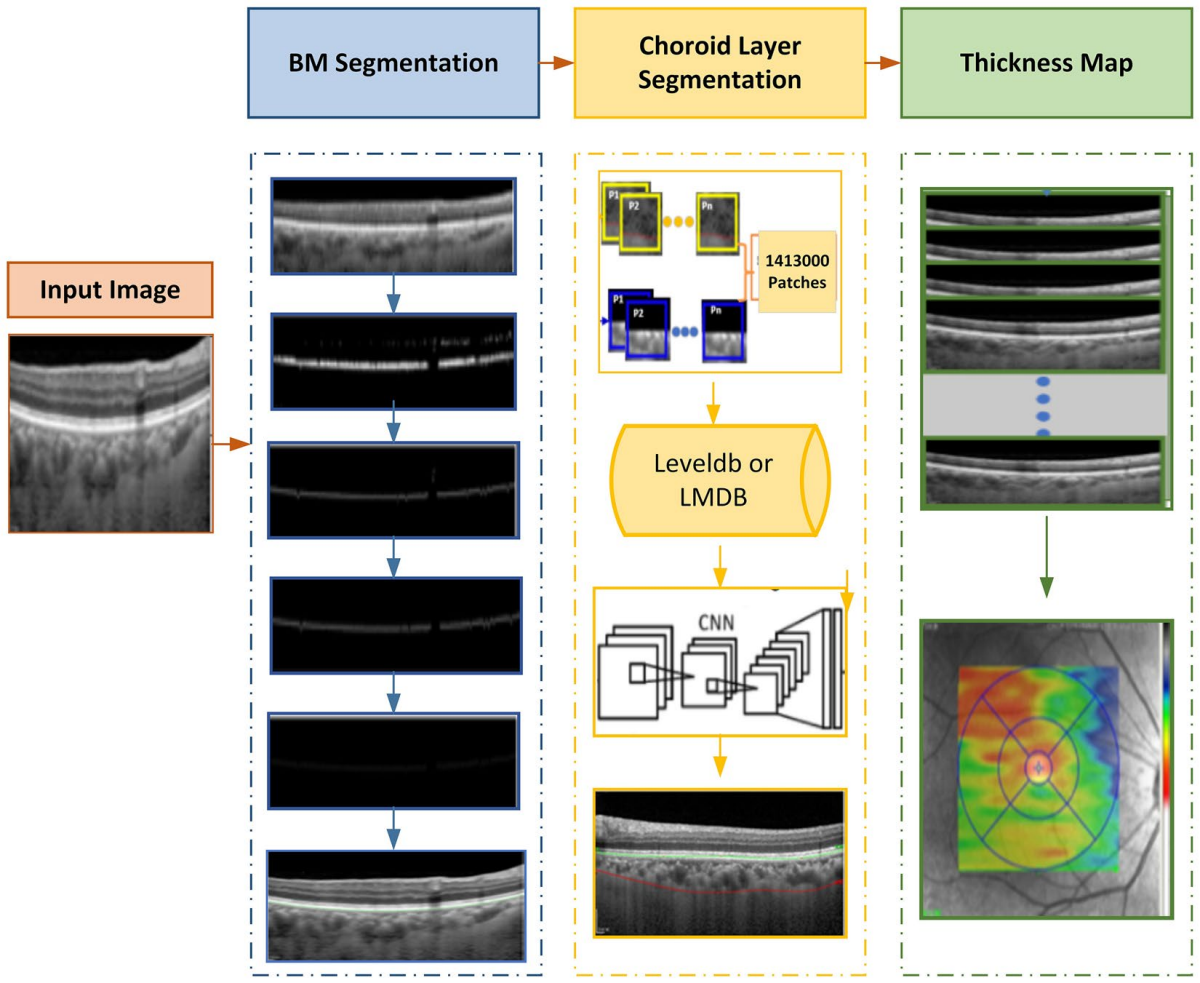
The schematic illustration of the proposed method, which is composed of three stages: BM segmentation, Choroid layer and thickness map generation.

The proposed methodology is comprised of 3 main modules including BM segmentation, choroid layer segmentation, and thickness map generation. Figure 4 depicts the overall approach of the proposed method. For the segmentation of the regions of interest, OCT images were taken as inputs to the system. The BM was segmented using a series of morphological operations whereas the segmentation of choroid layer, which required more image statistics, was extracted using a CNN. Later, thickness maps were generated using the extracted layers.

### Bruch’s Membrane Segmentation

**Figure 5:**
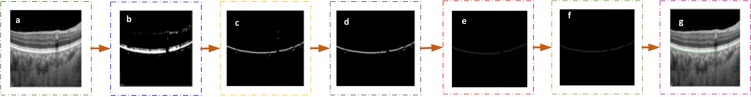
Bruch’s Membrane segmentation steps: In the diagram given above (**a**) represents the input image, (**b**) corresponds to the result of converting input image to binary format, (**c**) is the result thresholding operation, (**d**) represents the result of reconstruction (**e**) shows the result of thinning operation, and (**f**) step finally apply erosion and then spline fitting is applied to draw the final segmented BM area.

A series of steps were performed to carry out BM segmentation. If we analyze the OCT image, we can observe that BM segmentation is relatively easier than choroid layer segmentation. The reason is that the choroid layer has inhomogenous intensity and inconsistent texture, so it requires more image statistics to accurately segment. We first segmented the BM followed by segmentation of choroid layer. In this case, we can consider the segmented BM as a constraint while carrying out choroid layer segmentation. The segmentation of BM was performed using a sequence of morphological operations. Morphological operations mainly represent a collection of non-linear operations associated with the morphology or shape of the features in the OCT image. Morphological image processing follows the goal of eliminating all the defects and maintaining structure of the image. These operations are confident on the associated ordering of pixel values, rather than their numerical values, so they are utilized more on binary images. BM boundary represents a clear white boundary in the OCT image as compared to the other layers/structures in the image. Additionally, as it is evident from the OCT images, the intensities of the BM boundary are homogeneously distributed. The histogram peaks corresponding to BM are distinct from each other as well as from the background. The distinctive feature of the proposed method is that it ensures the homogeneous intensity distribution for the BM boundary. This helped to improve the visibility of delicate mass lesions and helped to reduce unwanted background information. A summary of the performed steps are shown in Fig. 5. The steps involved in this process are detailed as follows:

### Thresholding

The first step was to convert the image into binary and extract the region of interest. The reason behind this step was to extract the pixels containing the region belonging to the BM boundary within the image. In order to obtain an optimal threshold value, OCT images were tested using a range of threshold values. After the analysis, the optimal threshold value was set to 190. This step helped determine the foreground and background pixels of the image. The output of the step was binarized data containing a range of intensity values. The threshold was defined as:

1 $$(\begin{array}{cc}{p}_{(ij)}\in {S}_{1} & {\rm{if}}\,0\le {P}_{(i,j)} < {t}_{b}\\ {p}_{(ij)}\in {S}_{2} & {\rm{if}}\,{t}_{b}\le {P}_{(i,j)} < L-1\end{array}$$

where *p*_*i*,*j*_, *i* = 1, 2, …, *r*, *j* = 1, 2, …, *c* represents the pixel intensity of the image (with size r × c and n = L gray levels from zero to L-1), *t*_*b*_ is the set threshold, *S*_1_ and *S*_2_ are sets in which pixels with intensities between thresholds k and k-1 are located. Image was divided into two sets *S*_1_ and *S*_2_ (background and foreground pixels respectively) using a threshold at the level *t*_*b*_. Here *S*_1_ = [0, …, *t*_*b*−1_] and *S*_2_ = [*t*_*b*_, …, *L* − 1].

### Reconstruction

After acquiring the binary image, a reconstruction approach based on the morphological opening was applied to remove the noise and preserve the shape of BM layer that was not removed by the morphological erosion operator in the OCT image. The block analysis method can be assumed to be similar to this approach based on the fact the image is manipulated as a whole rather than dividing it into blocks. Firstly, a structuring element B of size 3 × 3 was formulated by considering minimum *I*_*min*_(*x*) and maximum intensity max *I*_*max*_(*x*). Later, the background benchmarks *R*_*i*_ were defined based on the obtained values. The background criterion was formulated as:

2 $$R(x)=\frac{{I}_{min}(x)+{I}_{max}(x)}{2}$$

where *I*_*min*_(*x*) and *I*_*max*_(*x*) represents morphological erosion and dilation respectively. Therefore,

3 $$R(x)=\frac{{\epsilon }_{\mu }(f)(x)+{\delta }_{\mu }(f)(x)}{2}$$

where ϵ represents the erosion operator followed by *δ* operation and *μ* represents the structuring element. The employed reconstruction approach yielded better results when compared to the traditional block cutting method as the used method provided better local analysis of the OCT image. Eight neighboring pixels at every point within the OCT images were considered by the structuring element *μ*.

### Thinning

After image reconstruction, the number of rows spanned by the objects were taken into account. In order to select the object-spanned rows, a range was defined, given that the rows contained most of the points. Any point outside the selected range was discarded. Then, foreground pixels not part of the BM boundary were eliminated. The morphological thinning operation was applied on the extracted rows containing the BM area in the OCT image. The purpose of this step was to iteratively remove pixels exclusive to the shape and to shrink it without shortening or breaking BM boundary apart. To achieve the desired goal, we observed whether an edge pixel *P*_1_ should be removed by considering its 8 neighbors in the 3 × 3 neighborhood. To delete or mark the pixels, the approach of connectivity numbers was employed. The approach helped us to find the number of objects being connected to a specific pixel in the OCT image. The connectivity number was calculated using the following equation:

4 $${C}_{n}=\sum _{k\in s}{N}_{k}-({N}_{k}\,\ast \,{N}_{k+1}\,\ast \,{N}_{k+2})$$

where *N*_*o*_ represents the central pixel and the color of eight neighbors is represented by *N*_*k*_. The pixel to the right of the central pixel is represented by *N*_1_. All remaining neighboring pixels were numbered in counter-clockwise order around the central pixel.

### Curve Fitting

The next step was to mark the boundary of segmented BM. This step was performed using spline curve fitting. Curve fitting was applied to find a function f(x) based on the data (*x*_*i*_, *y*_*i*_) where *i* = 1, 2, …, *n*. The residual was minimized by the function (x). The residual is the distance between the data samples and f(x). A better curve fitting can be obtained through a smaller residual. Spline fitting being applied for the curve fitting was calculated as:

5 $$f(x)={a}_{i}{(x-{x}_{i)})}^{3}+{b}_{i}{(x-{x}_{i})}^{2}+{c}_{i}(x-{x}_{i})+{d}_{i}$$

where *a*_*i*_, *b*_*i*_, *c*_*i*_ an *d*_*i*_ represents set of polynomial coefficients and *x*_*i*_ = 1, 2, …, *n* represents the data points to be mapped in the curve. The output of this step is the final segmented BM boundary. Figure 5 illustrates the output of each step being performed for the BM boundary segmentation; it has 7 subparts, each representing a result of the different morphological operations being applied on the input OCT image.

### Choroid Layer Segmentation Using Deep Learning

As discussed above, BM segmentation is easier when compared to the segmentation of the choroid layer. Based on the the aforementioned challenges and provisions, a deep learning approach was taken to carry out the desired segmentation.

**Figure 6:**
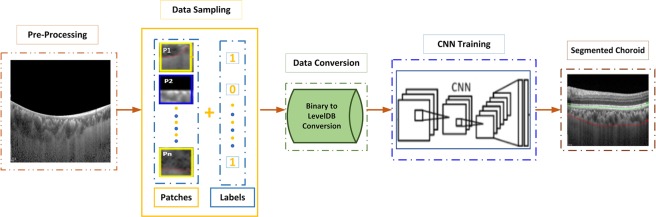
Steps for Choroid Layer Segmentation: Choroid layer was segmented using a series of operations, including pre-processing, data sampling, data conversion, CNN training and choroid layer segmentation.

Figure 6 illustrates the steps that constituted the segmentation process. These steps include pre-processing, data sampling, data conversion, CNN training and final choroid layer segmentation.

### Pre-Processing

In the pre-processing phase, the segmented BM was kept as a point. All points in the area above the reference point were set to black. This was to reduce the area to be processed for the segmentation of the choroid layer. The output of BM segmentation (the segmented layer) was fed as an input to this stage. Analysis of the OCT image showed that the interface between the sclera and choroid layer is inseparable in the majority of cases and, as a result, accurate segmentation of the choroid layer is a challenging task. Based on this analysis, the purpose of the pre-processing stage was to obtain the definite region of the OCT image containing the choroid layer. Consequently, the pre-processing step simply eliminated all of the area above the segmented BM.

### Data Sampling

The data provided by the doctors contained manually segmented BM and choroid layers. The segmentation of the required boundaries on OCT images was performed by the experts manually. The data sampling step made use of the provided data to sample the patches to be used for training of the CNN. The manually segmented images were sampled patch-wise from top to bottom and left to right. The purpose of sampling was to classify pixels as on-line or off-line patch. The patch size used in our research was 32 × 32. This patch size was used because:

- Very small patch sizes make it difficult to extract useful information from the region of interest
- Very large patch sizes may contain surplus information and increase the complexity of processing

**Figure 7:**
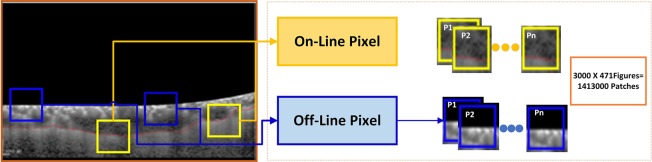
Patch Sampling Process: As the choroid layer was marked by a red curve, so any patch containing a pixel on the lower line (i.e. choroid layer) was classified as on-line 0r 1 whereas any patch containing no pixel on the lower line was classified as off-line or 0. The figure shows that the patches having no pixel on boundary are labeled 0 whereas patches having any pixel on line are labeled as 1.

As patched images were manually segmented by the ophthalmologist, the choroid layer was marked by a red curve. The classification of the patches was performed in order to pick the patches containing the choroid layer. The illustration of the patch labeling process is described in Fig. 7.

The stride between each patch was taken as 5 pixels. A threshold was then set to discard patches containing too many black pixels. If more than 1/3 of the patch contained black pixels, it was discarded. The data set contained images of 21 patients. Data of 11 individuals was used to train the model whereas data of 10 individuals was used to test the model. In order to balance labels 0 and 1, the patches of label 0 were randomly chosen to have the same number as label 1. There were almost 3000 patches for one figure, so the total number of patches being sampled from the data was about 1,575,000.

### Data Conversion

Next, we converted the data to binary format. The binary format for CIFAR-10 is shown in Equation 6. Here, the first image label (i.e. 0 or 1) was represented by the first byte. Image pixel values were denoted by the next 3072 bytes. Red channel values were indicated through the first 1024 bytes, the following 1024 bytes represented green channel values and the last 1024 bytes corresponded to blue channel values. Row major order was used to store these values, where an initial 32 bytes represented the red channel in the initial row of the image.

6 $$\begin{array}{ccc}\mathrm{ < 1}xlabel\mathrm{ >  < 3072}xpixel >  & \ldots \ldots \ldots  & \mathrm{ < 1}xlabel\mathrm{ >  < 3072}xpixel > \end{array}$$

The next step was to convert the binary file to leveldb format because caffe only supports leveldb or lmdb^51^. We used the program provided by Caffe to do this transformation. After the transformations, the CNN model was trained using the extracted information. After training the model it was used for the segmentation of the choroid layer.

### CNN Training

**Figure 8:**
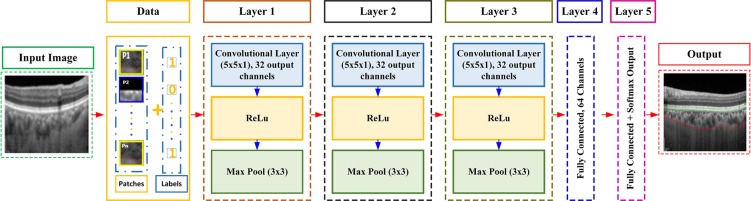
CNN Architecture: The CNN architecture being used in this research entails of a sequence of layers including convolution, rectified linear units followed by max pooling layer, fully connected and a softmax layer.

The CNN layering structure used in this work was the Cifar-10 model^52^. We used a pre-trained CNN Cifar-10 architecture to generate features and trained the network using sampled data. The CNN architecture was composed of a layering structure. The layers of the CNN included layers of convolution, pooling, and linear unit nonlinearities. There was also a linear classifier for contrast normalization on top of all these layers. The modification was carried out in the last layer of the CIFAR-10 model. The architecture of the CNN used in our approach is shown in Fig. 8.

Figure 8 shows the overall structure of the CNN model being used in this work. Because contrast of OCT images was low and the texture between the choroid layer and sclera was inhomogeneous, we preferred to carry out the segmentation through slices being sampled from the OCT images. Thus, our model processed each 2D OCT image sequentially in slices. The proposed approach predicted the class of every patch based on the associated label of the patch. The CNN processed the *M* × *N* patch centered on that pixel. Therefore the input to the CNN model was an M × N 2D patch. The CNN architecture’s main structuring element was the convolutional layer. In this case, numerous layers can be piled on top of each other to make a pyramid of image features. Each layer in the pyramid harvests features from the prior layer. A stack of input planes was fed to the convolutional layer of our model as its input. The convolutional layer processed the procedures that were being fed and produced feature maps as output. The feature maps were produced by applying spatially local non-linear feature extractors to all spatial neighbors of the input planes. As a result, we obtained an organized topological map of the responses. For the first convolutional layer, different OCT 2D M × N patches were represented through the individual input planes. In successive layers, the input planes were characteristically comprised of the feature maps of the preceding layer. The implemented model contained 5 layers starting with three convolutional layers followed by two fully connected layers. The final layer of the network was a softmax layer used for the final classification. The convolutional layers were followed by max pooling and Rectified Linear Units (ReLU). Kernels in the convolutional layer were of size 5 × 5 with a step size of 1. The kernels of max pooling layer were of size 3 × 3 with a step size of 2. The convolutional layer mainly performed the feature extraction phase - it helped translate the input to its equivalent characteristics.

The pyramid of complex features in the CNN model was considered by giving a convolutional layer’s output feature maps as input channels to the successive layer of convolution. In precise denotation, if focusing on a feature map, it represents a layer of neurons. Each neuron in the layer corresponds to a coordinate within the feature map. The size of the neuron’s separate field correlates to the size of the kernel. The connection among the neurons of the same layer and the previous layer is represented by the value (weight) of the kernel. In the learned kernel’s weights, each kernel is adjusted to a diverse orientation, spatial frequency and scale suitable for the training data. Finally, in order to obtain segmentation labels, we connected the convolutional hidden layer to a fully connected layer followed by a fully connected and softmax layer. As the layers were fully connected, there were 64 output channels because input channels were 32. Each kernel in the fully connected layer acted as the ultimate detector of the choroid from one of the segmentation labels. The purpose of this layer was to map activation volume from the blend of preceding different layers into a class probability distribution. The CNN was trained based on the extracted patches from the OCT images. Test image patches were used to get the overall test accuracy of the proposed model and develop an overall understanding of the image classification as a segmentation procedure for the choroidal boundary.

As discussed in the data sampling and data conversion sections of the methodology, the input OCT image was analyzed with a patch window being traversed on the OCT image. The window size was set to 32 × 32 and the patches were analyzed and given labels according to the contents contained within that window patch. The whole image was traversed and the image was cut into 32 × 32 image slices. As a result, we obtained many patches from a single OCT image. The OCT image was classified into two classes: part of the choroid layer or not part of the choroid layer. Therefore, all patches were classified into the two classes. The total sampled patches amounted to about 1,575,000 patches, extracted from 525 OCT images of 21 individuals. Data of 11 individuals was used to train the network, meaning 825,000 patches (275 images) were used to train the network. Data of 10 individuals was used to test the network, meaning 750,000 patches (250 images) were used.

**Figure 9:**
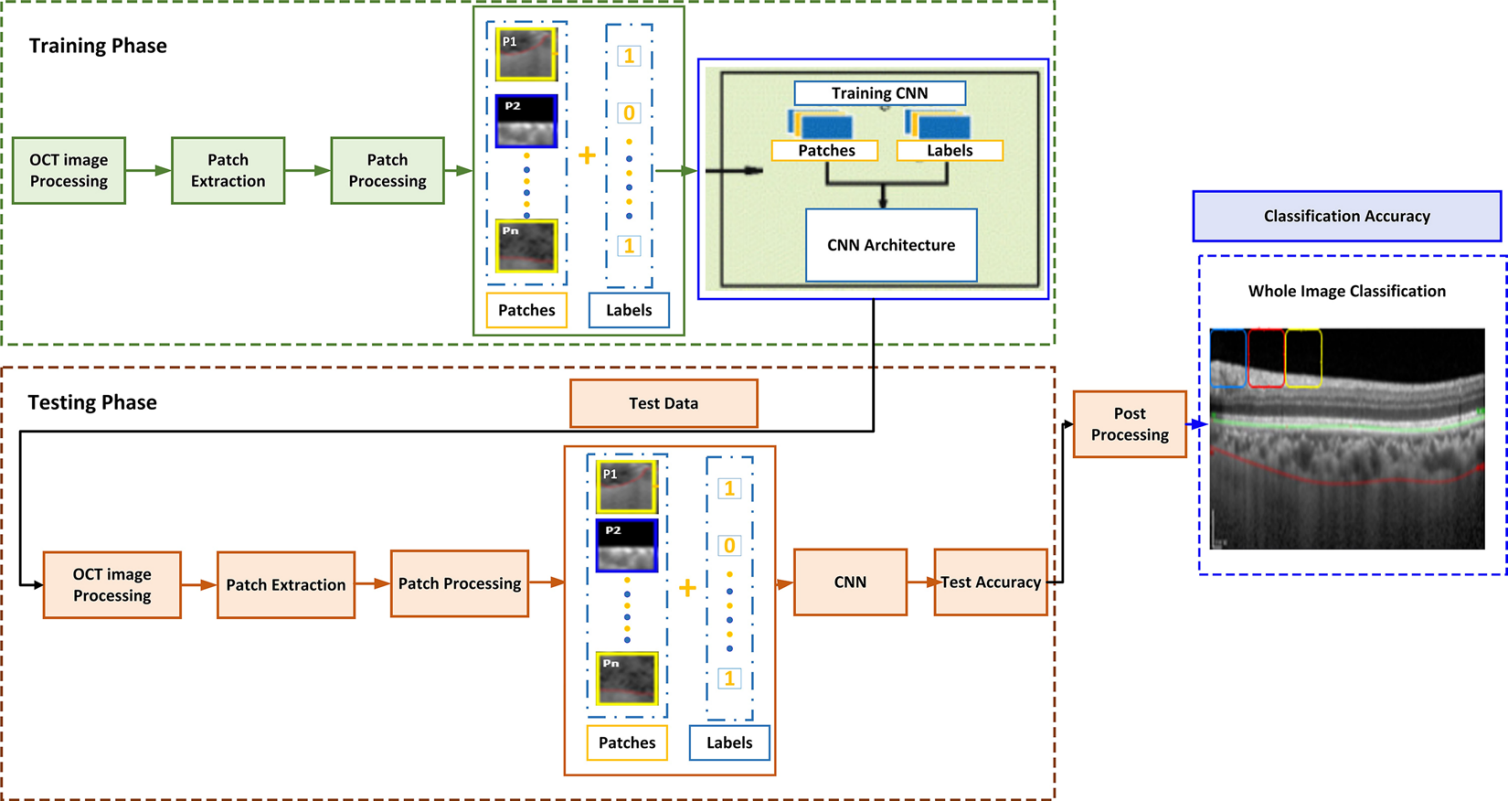
Overview of CNN training and choroid layer segmentation: the CNN was trained based on the extracted patches from the OCT images, test image patches were used to get the overall test accuracy of the proposed model and to obtain the overall image classification as segmentation of choroidal boundary.

After sampling patches from all the images, the sampled patches were used to train the model. Figure 9 shows the testing and training phase of the network. The distribution of the segmentation labels was obtained by analyzing the output of the convolutional network. The objective of the training condition was to lessen the negative log-probability from every OCT image by exploiting the likelihood of all labels in the training set being used. This was formulated as:

7 $$-Lo{g}_{max}\frac{Y}{X}=su{m}_{ij}-Lo{g}_{max}\frac{{Y}_{ij}}{X}$$

The concept of weight decay was applied for regularization of the learned variables. The sum of weight decay and cross-entropy was calculated to formulate the objective function of the network.

Based on the proposed methodology, the database that was used contained data as *D* = *X*, *Y* = *x*^*i*^, *y*^*i*^, *i* ∈ 1, …, *N*, where *x*^*i*^ represents the input patch and *y*^*i*^ is its corresponding label. As the patch can be one of the two categories, either part of the choroid layer or not, the considered problem was multi-categorical. In this case, every label *y*^*i*^ belonged to one of C categories, *y*^*i*^ ∈ 1, …, *C*. Likelihood over the training data D was maximized to find the parameters of the model with respect to the parameters *θ*:

8 $$\begin{array}{rcl}{\theta }_{\ast } & = & \mathop{{\rm{argmax}}}\limits_{\theta }=p(Y|X,\theta )\\  & = & \mathop{{\rm{argmax}}}\limits_{\theta }=p(Y|X,\theta )({y}^{1},{y}^{2},\ldots ,{y}^{N}|{x}^{1},{x}^{2},\ldots ,{x}^{N},\theta )\\  & = & \mathop{{\rm{argmax}}}\limits_{\theta }\mathop{\prod }\limits_{i=1}^{N}p({y}^{i}|{x}^{i},\theta )\end{array}$$

where *p*(*θ*) is the objective function being updated by the standard gradient descent, and *D* = *X*, *Y* = *x*^*i*^, *y*^*i*^, *i* ∈ 1, …, *N*, where *x*^*i*^ is an input image and *y*^*i*^ is its corresponding label. This meant that an anonymous independent and identical distribution (i.i.d.) was used to sample the training set. The basic purpose was to minimize the negative log-likelihood of the training data. This was formulated as:

9 $${\theta }_{\ast }=\mathop{{\rm{argmin}}}\limits_{\theta }-\sum _{i}\sum _{c}||{c}^{yi}[{f}_{c}({x}^{i},\theta )-log\sum _{j}{e}^{fj({x}^{i},\theta )}]$$

where *f* represents the monotonic function defined at the label, *y*^*i*^ belongs to one of categories *c* and *e* denotes the energy function over the monotonic function *f* and input data *x*_*i*_. The cross entropy of the real targets can be minimized based on these assumptions. Target distribution over the data set was carried out through one-hot encoding. Considering these assumptions the problem of minimization was formulated as:

10 $${\theta }_{\ast }=\mathop{{\rm{argmin}}}\limits_{\theta }-\sum _{j}[f{c}_{i}^{\theta }({x}^{i},\theta )-log\sum _{j}{e}^{fj({x}^{i},\theta )}]$$

Here one-hot encoding of the training data is represented by ${c}_{i}^{\theta }$. An iterative approach was used to find the minimum, i.e. the loss function was minimized in order to estimate the parameters *θ* of the network. The criterion was defined as:

11 $$L(\theta ,X,Y)=-\sum _{i}[f{c}_{i}^{\theta }({x}^{i},\theta )-log\sum _{j}{e}^{fj({x}^{i},\theta )}]$$

where *L* represents the loss function computed over objective function *θ*, the input patch is *X*_*i*_ and its associated label is *Y*_*i*_. Stochastic approximation of the gradient was applied for optimization of the network. In this case, a random training sample *x*^*i*^, *y*^*i*^ was used to approximate the gradient. The parameters of the network were updated as:

12 $${\theta }_{t+1}\leftarrow {\theta }_{t}-\eta {\nabla }_{\theta }L(\theta ,X,Y)$$

where *η* represents the learning rate. Each different learning rate for every parameter *θ*_*t*_ is represented as *θ*_*t* + 1_ and ▽ is the gradient of the cost function. Mini-batch gradient descent was then used to optimize the loss function. The optimization was achieved through the midterm among the batch method and stochastic. The approach made use of a subset of *n* < |*D*| training data to update the parameter of the network. This was formulated as:

13 $${\theta }_{t+1}\leftarrow {\theta }_{t}-\eta {\nabla }_{\theta }L(\theta ,{X}^{i,\ldots ,i+n}),{Y}^{i,\ldots ,i+n})$$

After training the model, it was used for the segmentation of the choroid layer. In order to segment out the choroid layer, a counter matrix was proposed. The matrix was created with the same size as the image and initialized as a zero matrix. After each prediction of a patch in terms of its label, if the label was 1, all the pixels in the counter matrix corresponding to the patch were increased by 1. After finishing the prediction, the counter with the highest number in each column was chosen to fit in the cubic curve for the choroid layer segmentation.

**Figure 10:**
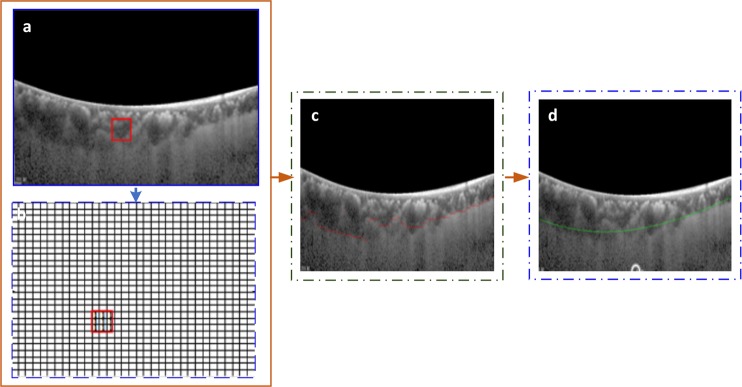
Counter Matrix Concept: (**a** and **b**) represents the counter matrix, (**c**) calculate the maximum index in each column and (**d**) shows the segmented choroid layer after applying polynomial fitting.

**Figure 11:**
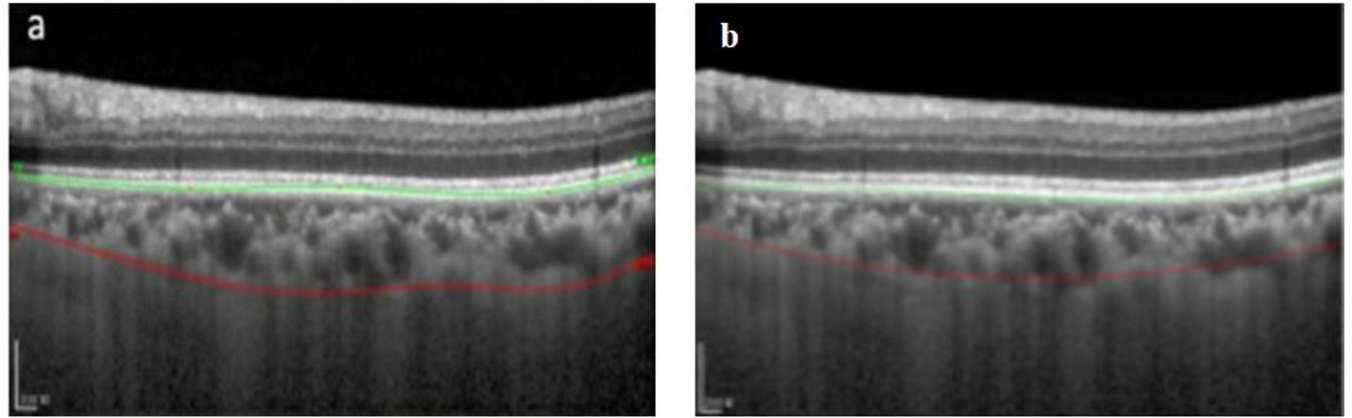
Result of Choroid Layer Segmentation using deep learning method: (**a**) shows part of the image containing an OCT image being labeled by the doctor, where BM is marked in green and choroid is marked in red. (**b**) Represents the OCT image segmentation performed by the proposed methodology, here the choroid layer is marked in green and BM is labeled in red color.

Figure 10 shows the concept of the counter matrix, where every patch was analyzed based on its label and, if the label was 1, all pixels corresponding to that patch in the counter matrix were set to the value 1. Later, in order to mark the boundary of the choroid layer, the counter having highest number in every column was selected to be fit in the curve for the marking of the boundary. Figure 11 shows the comparison between the OCT image labeled by specialists and OCT image segmented by the proposed method.

### Thickness Map

**Figure 12:**
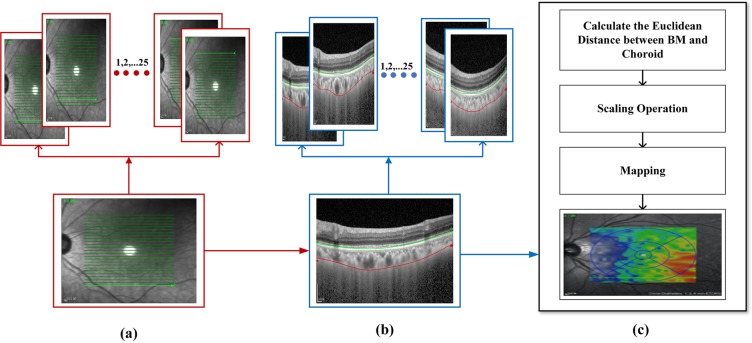
Choroid layer slices to calculate Thickness Map: The thickness of each image was taken into account in order to generate the thickness map of each individual. As a result of processing each image we get a matrix representing thickness of every layer. Finally in order to get the map, matrix was resized to actual image size in order to draw the map.

As the focus of this research was to measure the choroidal thickness for the analysis of the health of the retina, thickness maps were generated following segmentation of BM and choroid layers. Thickness maps corresponding to each individual was generated based on the segmentation being performed. As each individual had 25 OCT scans, representing a different depth of choroid, all 25 images were considered to generate the thickness map. The thickness map can be defined as the Euclidean distance between the two surfaces: BM and choroid layers. In order to measure the required distance, the boundary of BM was taken as a reference boundary for the entire choroid region including the choriocapillaris. The distance between the BM and the upper surface of the choroid layer represents the choriocapillaris distance. Finally, in order to calculate the choroidal thickness, the distance between the BM and the lower surface of the choroidal vasculature was calculated. Choroidal vasculature and BM-equivalent thickness maps were created for all subjects. The error rate between the thickness map generated by doctors and map generated by the proposed method was also calculated. Figure 12 illustrates how the thickness of choroid layer was calculated, with specific steps described below:

- For each figure, the width was 760 pixels and the height was 456 pixels
- Next, a scaling operation was applied. We imposed 200 um maps, with 25 pixels in width and 76 pixels in height, so we could get the true thickness of each point
- There were 25 figures for each patient and the interval between two figures was 240 um
- According to steps 1–3, we could map a 25 × 760 matrix (refers to the pixel value on the figure) to 5760 um × 6080 um (refers to the real value of patient). The thickness value would then be mapped in different colors in the generated map.

## Experimental Analysis

### Dataset

The data set of the ocular images used in this work was collected from Shanghai Jiao Tong University Affiliated Sixth People’s Hospital, China by using the volume scan mode with swept source OCT. The task of collecting data was undertaken in agreement with the organization’s research ethics conventions. The study was performed in accordance with the Declaration of Helsinki(DoH) and approved by the Ethics Committee of Shanghai Sixth People’s Hospital (reference: 2014-11-01). Written consent has been obtained from all subjects, and informed consent for study participation has been obtained. The data set contained 525 OCT scans from 21 healthy subjects. The images were centered at the macula region. All 21 subjects included 25 OCT scans showing different depths of the macula region. 275 images were used to train the network whereas 250 images were used to test the methodology. The OCT scans of each subject were uniformly selected and manually labeled along the outermost edges of the choroidal and BM boundaries. 25 OCT scans of every individual were examined using Heidelberg Eye Explorer Software (Heidelberg Engineering, Heidelberg, Germany).

The examined data provided (i) the automated retinal boundary for the choroidal boundary, (ii) the dots on the internal limiting membrane to the RPE boundary, and (iii) the marks on the RPE boundary to the choroidal scleral junction. Choroidal Volume (CV) was also calculated at the 6-mm circle. Out of 21 individuals, data of 11 people had normal OCT B-Scans, 4 OCT B-Scans were from individuals affected by short sightedness, 3 individual’s data were affected by glaucoma and another 3 patients were suffering from mild DME. For each image, an expert ophthalmologist from Shanghai Hospital 6 manually annotated 2 boundaries (BM and the choroid layer). To overcome and reduce the manual segmentation error, two expert ophthalmologists provided the segmentation measurements of the required boundaries. Results between the two experts did not differ significantly (p = 0.359). The average of the two results was used in the conducted study. The manual segmentation by the experts was used as ground truth. Variation between slices (different depth of the choroid) was relatively small around the macula area; this interpolated ground truth was confirmed as appropriate by the experts. Table 1 can be analyzed to analyze the data division for the testing and training of the method.

**Table 1:** Data Distribution for testing and training, Total Patients: 21, data of 11 people was used for training and data of 10 individuals was used as testing.

Data	Normal	Short Sightedness	Glaucoma	DME
Total Patients	11	4	4	3
Testing	6	2	1	1
Training	5	2	2	2

### Ground Truth Labeling

Images being used in this research were from real patients. Ground truth was specified by the ophthalmologist of Shanghai Jiao Tong University Affiliated Sixth People’s Hospital. The specialist manually segmented BM and choroid layer on the OCT images. Thus, these manually segmented images were used as ground truth in the proposed method.

### Experimental settings

Our algorithm was implemented in MATLAB R2016b and run on a server with a GPU of 12 with 2 GB memory, with Intel(R) Xeon(R) CPU E5-2630 v4 @ 2.20 GHz (10 cores) processor, 64 GB of RAM space and an operating system of 64 bits. Cross entropy loss function was used following a pixel-wise soft-max over the network’s final output. The learning rate of the CNN was selected to be 0.001 for the first 20 epochs and 0.0001 for the last 20 epochs (total of 40 epochs). The system was trained with a weight decay of 0.0005 and a momentum of 0.9. In our trials, exploring further epochs did not significantly reduce the training error, but instead improved computational time. Because the system was convergent after 40 epochs, the model was not further trained. The Stochastic approximation of the gradient for the optimization was performed in mini batches of a size of 50 slices spliced from the training B-Scans with augmentation. The segmentation time for each OCT volume (25 slices) was 5 s−0.2 seconds per OCT B-scan.

### Evaluation Metrics

In order to test the results, some error calculation matrices were used to compute the error rate on the test data set. The metric was proposed in order to calculate the average error rate in terms of pixel values i.e. the average difference between the doctor’s segmented image and the result of our proposed method. The error rate was calculated as:

14 $$er{r}_{1}=\frac{\Vert \overrightarrow{A}\overrightarrow{B}\Vert }{h\ast w}$$

where $\Vert \overrightarrow{A}\Vert ={A}_{1}^{2}+{A}_{2}^{2}+\cdots +{A}_{2}^{n}$, $\overrightarrow{A}$ is the computed thickness vector, and $\overrightarrow{B}$ is the thickness vector provided by the ophthalmologists. *h* and *w* represent the height and width of the image, respectively. Another metric was proposed to calculate the average error between the thickness map generated by doctor and the thickness map generated by the proposed methodology. The metric can be defined as:

15 $$\begin{array}{cc}er{r}_{2}=\frac{|\bar{A}-\bar{B}|}{h} & where\,\bar{A}=\frac{{A}_{1}+{A}_{2}+\cdots +{A}_{w}}{w}\end{array}$$

**Figure 13:**
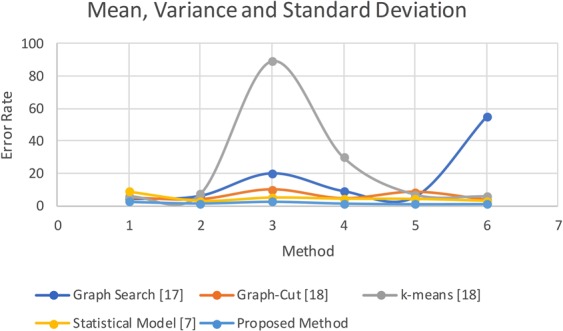
Comparison of Mean Error rate, Variance and Standard Deviation. The Comparison of Proposed method is made with the methods *k*-means^16^, Graph Cut^16^, Graph Search^19^ and Statistical Method^11^.

This metric was used to calculate the error rate of the proposed algorithm. Table 2 represents the average mean, variance and standard deviation calculated by the proposed methodology on the test data set. The term err1 represents the error rate between the doctor’s segmented image and segmentation performed by the proposed algorithm and the term err2 represents the error rate between the thickness map generated by the doctors and thickness map generated by the proposed method. Figure 13 shows the error rate computed on the test data set. Computed results are presented in Table 2.

**Table 2:** Mean, Variance and Standard deviation of the proposed method.

Error Type	Mean		Variance		Standard Deviation	
	err 1	err 2	err 1	err 2	err 1	err 2
Proposed Method	2.80	1.60	2.90	1.51	1.05	1.12
*k*-means^16^	6.03	7.27	89.26	30.25	7.21	6.04
Graph Cut^16^	5.04	4.02	10.25	4.59	8.72	3.71
Graph Search^19^	6.27	5.87	24.5	15.80	4.94	3.97
Statistical Model^11^	8.80	3.21	5.39	4.90	4.40	3.24

**Figure 14:**
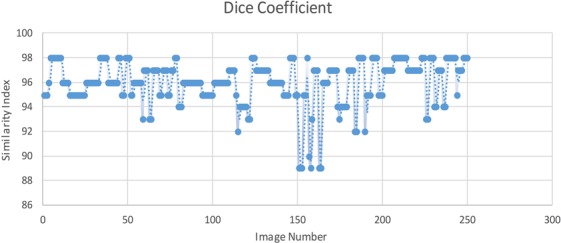
Dice Coefficient Similarity Results: representing the segmentation result similarity of the segmented choroid layer with the ground truth on randomly selected 25 images.

After calculating the error rate, dice coefficient was also created to measure the similarity between the segmentation result of the proposed method and the ground truth. The dice coefficient was computed on the test data set. The coefficient was calculated as:

16 $$D=\frac{\mathrm{2|}A\cap B|}{|A|+|B|}$$

where A and B are the segmented choroidal region and the manually labeled choroidal region, respectively. Figure 14 shows the similarity measures observed between the manual segmentation on the test data set and segmentation performed by the proposed method. It was found that the average of the dice coefficients over 250 tested images was 97.35% with a standard deviation of 2.3%, showing good consistency between manual labeling and segmentation results of our algorithm.

Results of the proposed algorithm shows consistency with the ground truth. Observation of the manually segmented images illustrates the fact that the input points are limited and thus represents a smoother boundary whereas the proposed algorithm monitors the valley pixels more narrowly. The error rates were observed to be higher when the choroidal region was thinner. Thus, the similarity measurement through dice’s coefficient resulted in smaller similarity for the thinner choroid. It is notable that the manually segmented choroidal-scleral boundary is paralleled to automated segmentation. As the choroidal-scleral contains many small curvature deviations, specialists are required to mark surplus points as well as the corresponding boundaries. These curvature deviations are not clearly visible unless the images are zoomed closely while performing the manual labeling. A more precise approach is adopted by the automatic segmentation through considering the small bumps and gaps present in the choroidal-scleral boundary. Thus the inconsistency between the automatic and manual segmentation is minimal.

**Figure 15:**
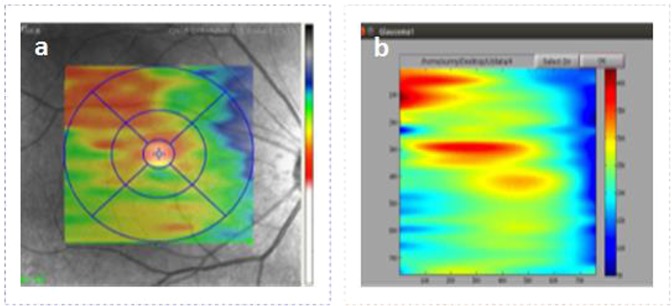
Thickness Map Comparison, Part (**a**) represents the thickness map generated by doctors segmented image where as part (**b**) corresponds to the thickness map generated by the proposed method.

The visual results of thickness map generated by our method and the doctors’ segmented images can be seen in Fig. 15. The results were then compared with other existing methods for choroid segmentation.

**Figure 16:**
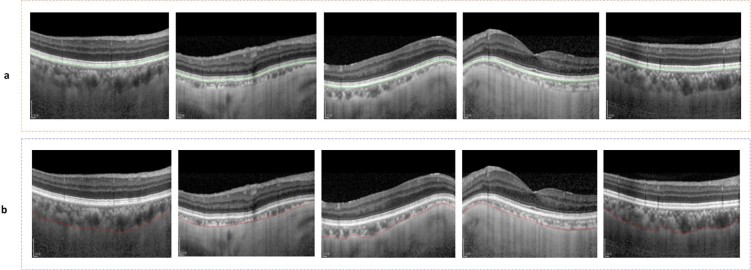
Sample results of BM and Choroid layer segmentation: (**a**) represents sample results of BM segmentation and (**b**) represents sample results of Choroid layer segmentation.

It is important to note that choroidal thickness measurements are highly variable in practice owing to the lack of a standardized definition of the choroidal-scleral junction and the often obscure imaging appearance in this region^8^. In our experiments, the outermost edges of the choroidal vessels are labeled as the posterior choroidal boundary and sample results of the choroid layer and BM are shown in Fig. 16.

The proposed method has been compared with other state-of-the-art methods as well. Results were computed on the test data set. The outcome of the comparison of the proposed method with a few of those state-of-the-art methods is shown in Table 3.

**Table 3:** Mean Error Comparison of choroid layer segmentation and thickness map with other state-of-the-art methods.

Method	Mean Error Rate (Choroid)	Mean Error Rate (Thickness)
Ground truth vs Graph Search^19^.	4.19	4.62
Ground truth vs Graph Cut^16^	5.04	5.28
Ground truth vs *k*-means^16^	6.03	6.47
Ground truth vs Statistical Model^11^	8.88	10.13
Ground truth vs Proposed Method	2.80	0.65

For further validation purposes, for each boundary segmentation, the mean signed and unsigned boundary positioning errors were compared with other algorithms such as graph cut, *k*-means and methods of^11,19^. Results are presented in Table 4 for each boundary. We implemented each of these algorithms and tested them on the test data set. In the *k*-means algorithm, we selected *k* = 3 and applied *k*-means algorithm on the image directly. In graph cut method, we used the result of *k*-means algorithm to create an initial prototype for each class and the distance between each image pixel to initial class was also calculated by *k*-means algorithm. The evaluation was performed on the same database and with the manually labeled ground truth. The experimental results show that the proposed method performed better than other states of art methods^53–56^.

**Table 4:** Signed and Unsigned Mean Error Rate Comparison (mean ± std).

Method	Signed Mean Error Rate		Unsigned Mean Error Rate	
	BM	Choroid	BM	Choroid
Ground truth vs Proposed Method	0.43 ± 1.01	2.8 ± 1.50	1.39 ± 0.25	2.89 ± 1.05
Ground truth vs Graph cut^16^	3.94 ± 0.67	40.70 ± 5.58	4.41 ± 1.79	40.50 ± 5.54
Ground truth vs *k*-means^16^	5.23 ± 2.45	−21.23 ± 10.21	6.43 ± 3.16	25.23 ± 8.72
Ground truth vs Graph Search Theory^19^	0.59 ± 1.28	8.24 ± 4.31	3.79 ± 0.94	9.28 ± 3.21
Ground truth vs Statistical Model^11^	0.78 ± 1.35	7.23 ± 4.31	2.84 ± 1.02	8.88 ± 4.40

**Figure 17:**
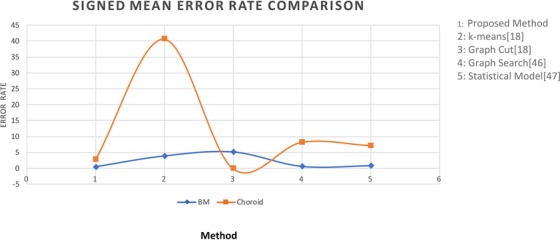
Signed Mean Error Comparison: The Comparison in terms of Signed Mean Error of the Proposed method is done against the methods *k*-means^16^, Graph Cut^16^, Graph Search^19^ and Statistical Method^11^.

**Figure 18:**
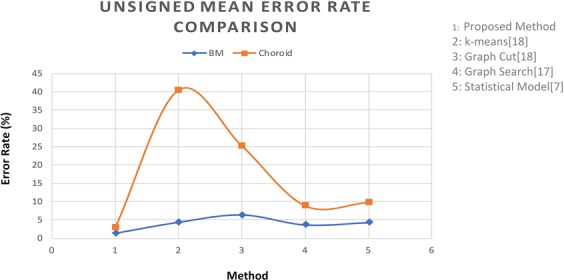
Unsigned Mean Error Comparison: The Comparison in terms of Unsigned Mean Error of the Proposed method against the methods: *k*-means^16^, Graph Cut^16^, Graph Search^19^ and Statistical Method^11^.

According to Table 4, observed signed border positioning errors were 0.43 ± 1.01 pixels for BM extraction and 2.80 ± 1.50 pixels for choroid segmentation, and the unsigned border positioning errors were 1.39 ± 0.25 pixels for BM extraction and 2.89 ± 1.05 pixels for choroid segmentation. Figures 17 and 18 show the mean signed and unsigned error observed on the tested data set, respectively, the signed and unsigned error were calculated with respect to the ground truth. The magnitude of signed error is much smaller than for unsigned error; hence, using signed error would misrepresent the level of deviation between the output and ground truth. Therefore, the unsigned error is reported in studies as a comprehensive measure of localization errors. The errors between the proposed algorithm and the reference standard were similar to those computed between the ground truth. The border positioning errors of the proposed method showed significant improvement over other algorithms, compared in both of the tables above.

Dice coefficient similarity measures and signed and unsigned border positioning errors of the proposed technique exhibited substantial improvement over other state-of-the-art methods. Results confirmed significant improvement through the proposed method. For instance, *k*-means and graph cut algorithm error rates are comparatively high when compared to the proposed methodology. Our method is also highly robust, overcoming many segmentation challenges posed by tissue anomalies such as drusen and RPE discontinuities to a large extent, and by low-quality images. This is reflected in the high number of correctly segmented images and the error rate that was observed. Validation against manual tracing demonstrated that the segmentation accuracy performed at least as well as specialists.

The role of deep retinal layers in the analysis of the progression of several diseases is becoming the focus of a notable number of studies. Besides the choroidal thickness measurement, the analysis of these layers opens the door to an array of global and local retinal feature descriptors that might be associated with ocular anatomy and functioning, which are still available to be explored. These functional and anatomical features can be highlighted with the help of precise tools for description of the choroid.

## Conclusion

In this paper, we have proposed and implemented a deep learning approach to automatically segment out the choroid layer. OCT image segmentation was carried out using morphological operations and convolutional neural networks. Analysis of OCT images showed that the BM boundary is easy to extract when compared to the choroid layer, so BM was segmented first using a series of morphological operations. The appearance of choroid layer is inhomogeneous, so more image statistics were required for accurate segmentation, and a CNN model was used. As the focus of this research was to analyze the health of retina based on the segmented boundaries, the thickness of segmented layers was computed to analyze the presence of retinal abnormalities. Data for real patients was used to test the results of the proposed method. The results showed an accuracy of about 97 percent. The acquired segmentation results were perceived as quite comparable to the segmentation performed by specialists. Results showed that the proposed method maintained reduced error rates to a great extent as compared to some other existing state-of-the-art methods. To further improve the precision of the proposed method, we will work on the following related topics: improving present techniques on 2D images, improving compatibility with 3D images, optimizing parameters by considering different patch sizes, optimizing stride between the extracted patches, and hopefully widening the scope of the work observe other retinal abnormalities including macular edema, hypertensive retinopathy, and glaucoma.

## Supplementary information

Sample Results

## Data Availability

The data sets generated during the study are available from the corresponding author on reasonable request.
