# Publisher Correction: Automatic Choroid Layer Segmentation from Optical Coherence Tomography Images Using Deep Learning

**DOI:** 10.1038/s41598-019-48368-x

**Published:** 2019-12-13

**Authors:** Saleha Masood, Ruogu Fang, Ping Li, Huating Li, Bin Sheng, Akash Mathavan, Xiangning Wang, Po Yang, Qiang Wu, Jing Qin, Weiping Jia

**Affiliations:** 1 Department of Computer Science and Engineering, Shanghai Jiao Tong University, Shanghai, 200240 China; 2 J. Crayton Pruitt Family Department of Biomedical Engineering, University of Florida, Gainesville, FL 32611 USA; 3 Faculty of Information Technology, Macau University of Science and Technology, Macau, 999078 China; 4 Shanghai Jiao Tong University Affiliated Sixth People’s Hospital, Shanghai, 200233 China; 5 Department of Computer Science, Liverpool John Moores University, Liverpool, L3 3AF UK; 6 Centre for Smart Health, School of Nursing, The Hong Kong Polytechnic University, Hong Kong, 999077 China

Correction to: *Scientific Reports* 10.1038/s41598-019-39795-x, published online 28 February 2019

In the Sample Results file originally published with this Article, the Affiliations of the authors were incorrect. These errors have been corrected in the Sample Results file that now accompanies the Article.

