# Supplementary material for: Automatic Choroid Layer Segmentation from Optical Coherence Tomography Images Using Deep Learning
Source: Sci Rep. 2019 Feb 28;9:3058. doi: 10.1038/s41598-019-39795-x (PMC6395677; doi:10.1038/s41598-019-39795-x)
Supplement: Supplementary file 1 — Sample Results [file 41598_2019_39795_MOESM1_ESM.docx]

**Automatic Choroid Layer Segmentation from Optical Coherence Tomography Images Using Deep Learning**

**Saleha Masood^1,+^, Ruogu Fang^2,+^, Ping Li^3,+^, Huating Li^4,+^, Bin Sheng^1,*^, Akash Mathavan^2^, Xiangning Wang^4^,**

**Po Yang^5^, Qiang Wu^4,*^, Jing Qin^6^, and Weiping Jia^4^**

^1^Department of Computer Science and Engineering, Shanghai Jiao Tong University, Shanghai, 200240, China

^2^J. Crayton Pruitt Family Department of Biomedical Engineering, University of Florida, Gainesville, FL 32611, USA

^3^Faculty of Information Technology, Macau University of Science and Technology, Macau, 999078, China

^4^Shanghai Jiao Tong University Affiliated Sixth People’s Hospital, Shanghai, 200233, China

^5^Department of Computer Science, Liverpool John Moores University, Liverpool, L3 3AF, UK

^6^Centre for Smart Health, School of Nursing, The Hong Kong Polytechnic University, Hong Kong, 999077, China

^+^Saleha Masood, Ruogu Fang, Ping Li, and Huating Li contributed equally

^*^shengbin@sjtu.edu.cn, wyansh@163.com

| **Manual Segmentation** | **Proposed Method Result** |
| --- | --- |
| Position  Thickness  choroid  BMosition  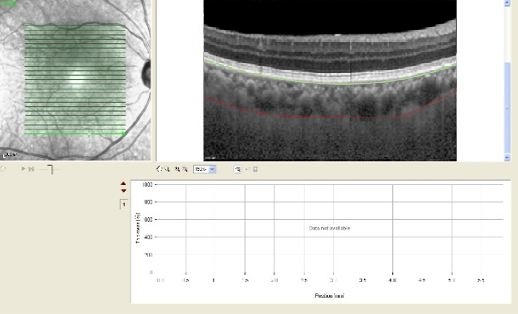 | 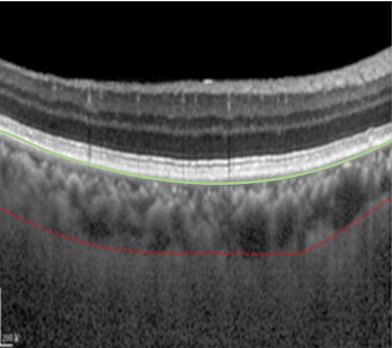  choroid  BMosition |
| 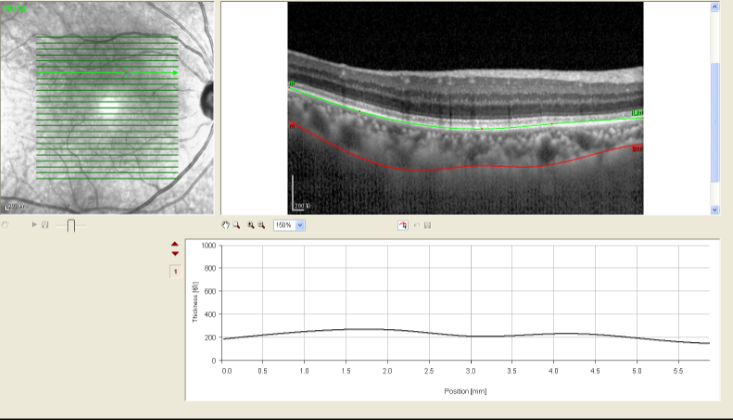 | 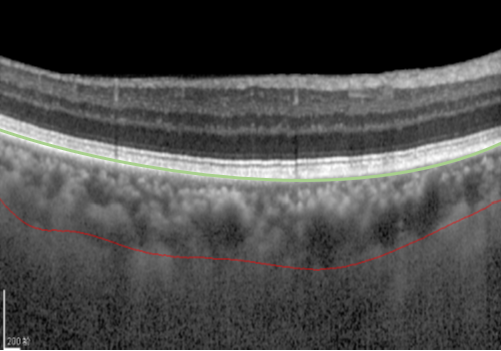 |
| **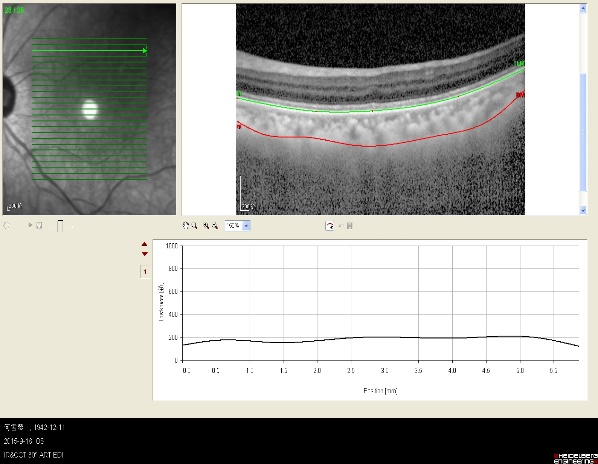** | 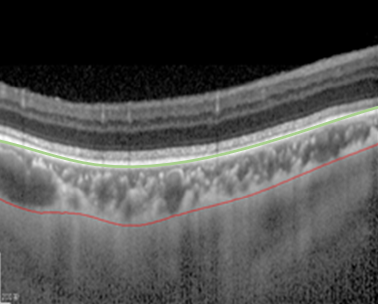 |
| **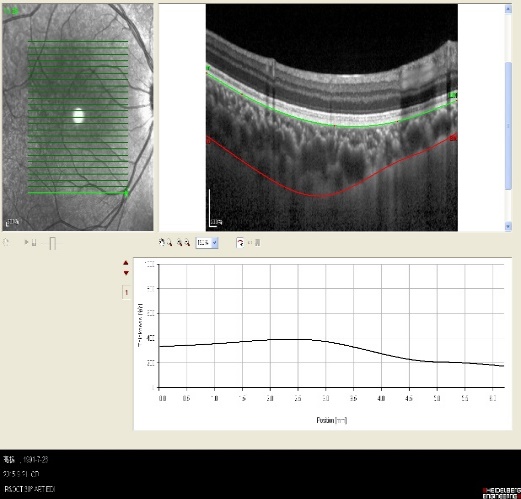** | 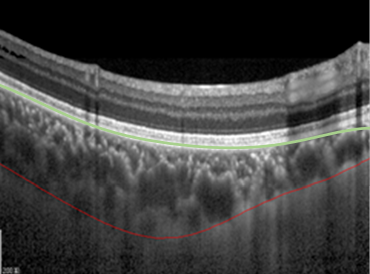 |
| **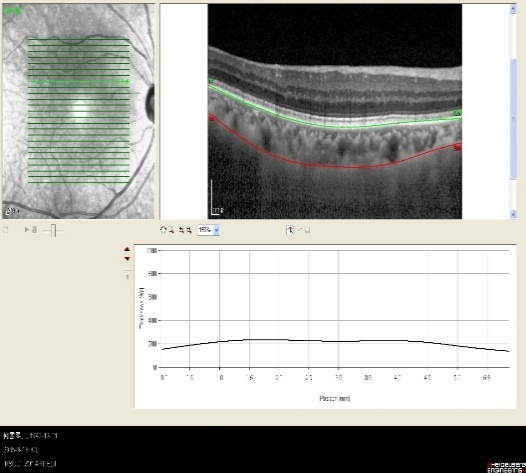** | 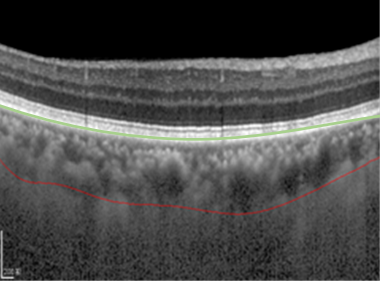 |
| 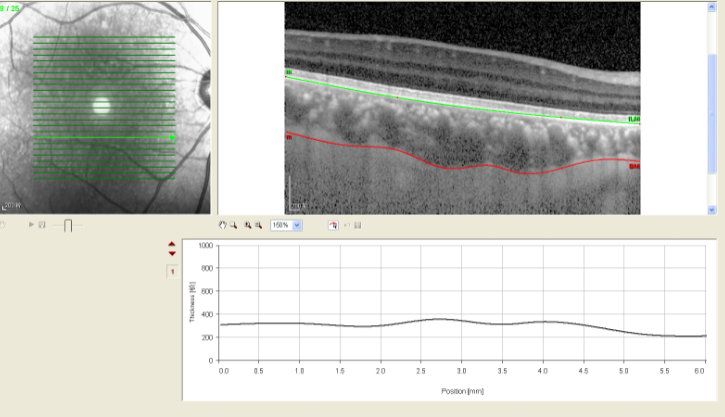 | 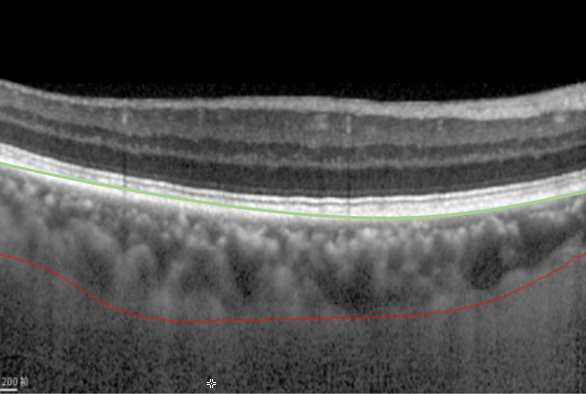 |
| **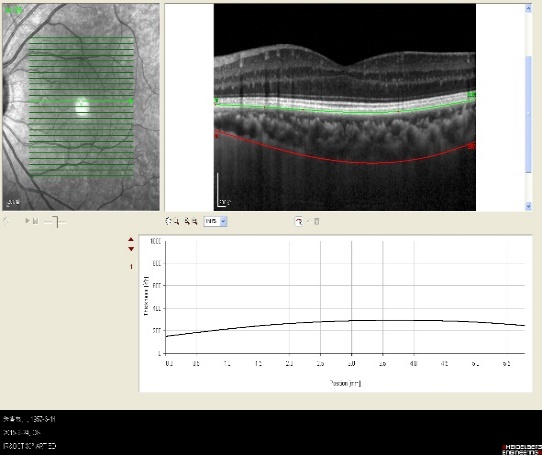** | 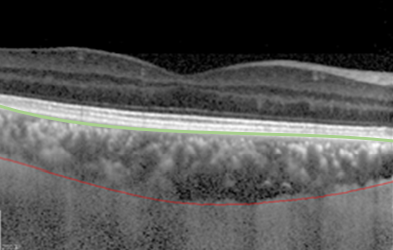 |
| **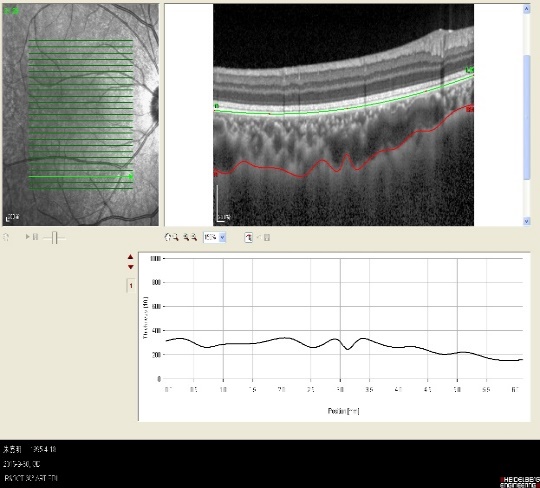** | 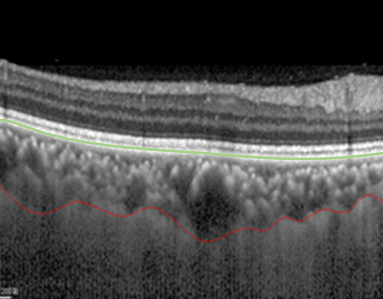 |
| **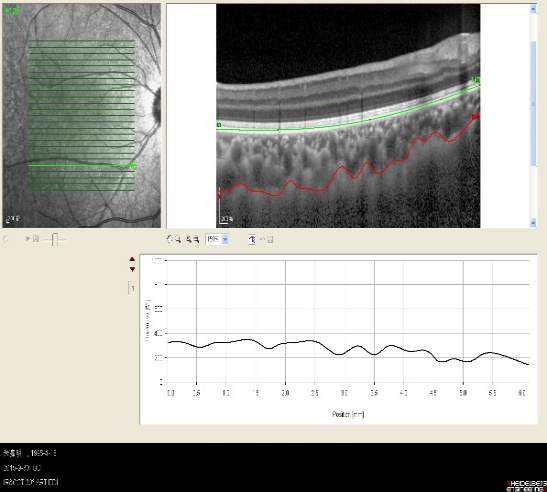** | 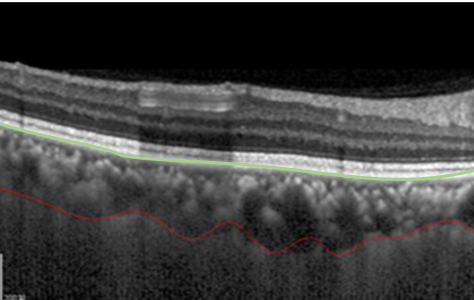 |
| **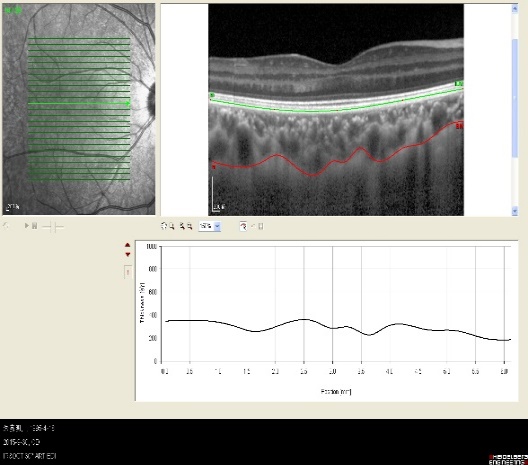** | 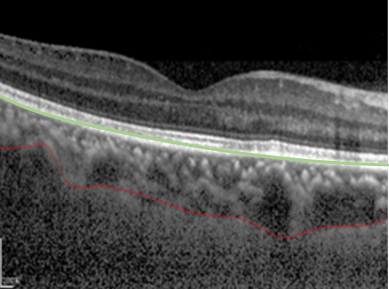 |
| **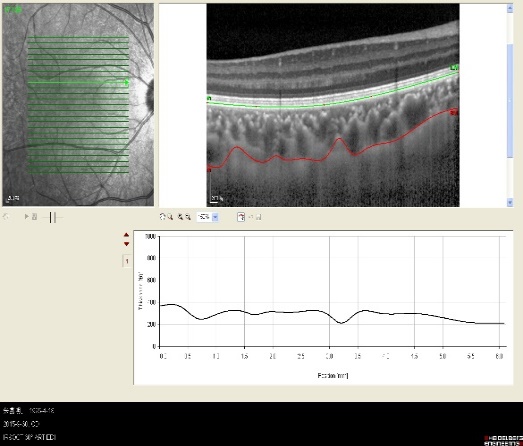** | 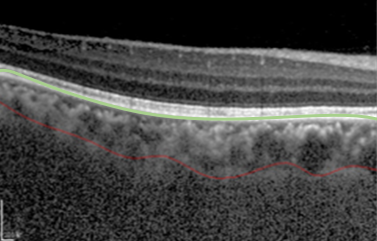 |
| **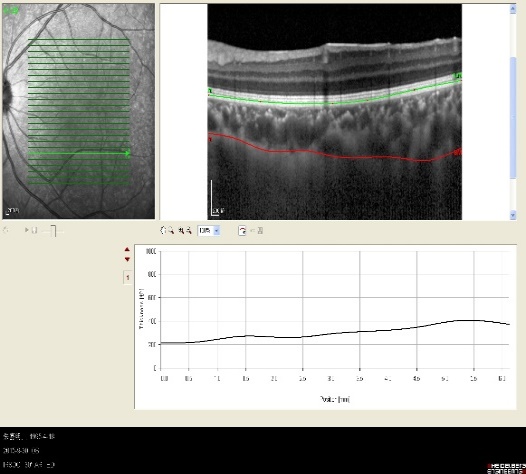** | 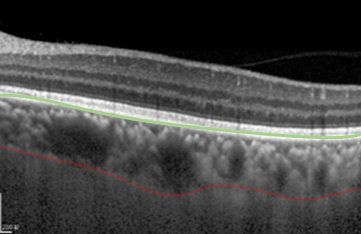 |
| **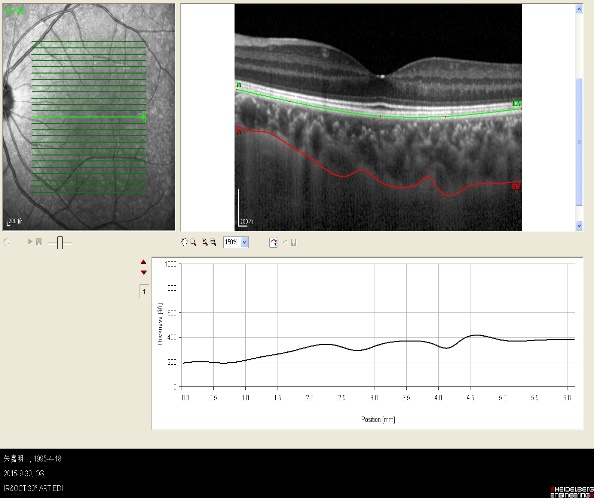** | 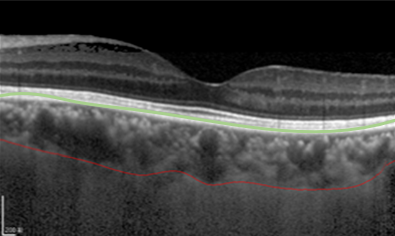 |
| **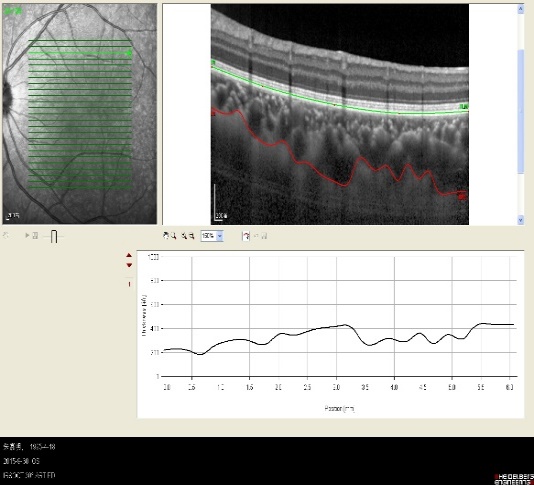** | 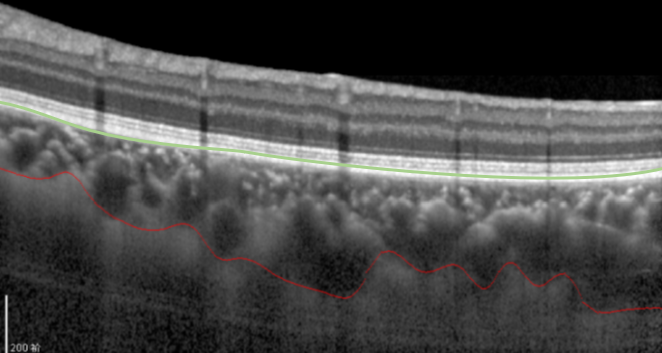 |
| **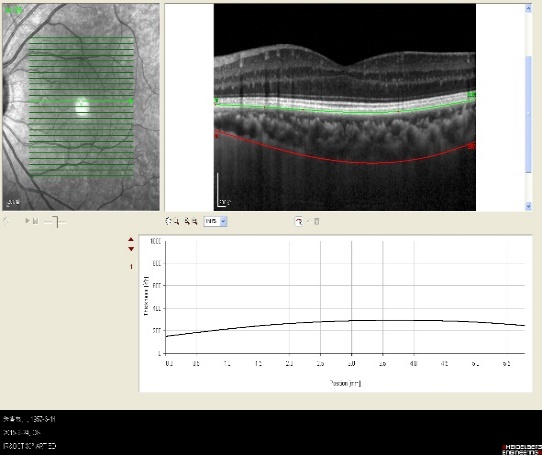** | 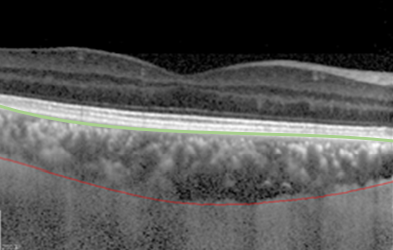 |
| **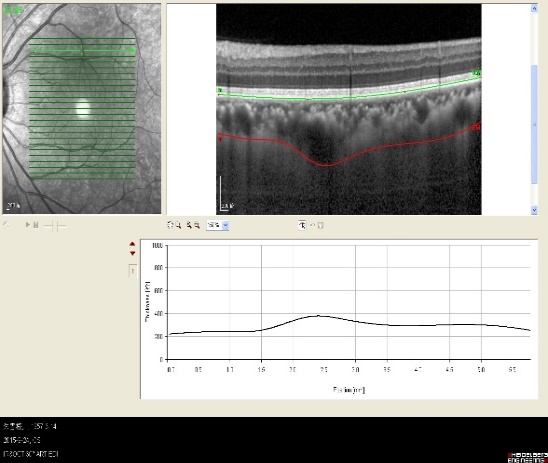** | 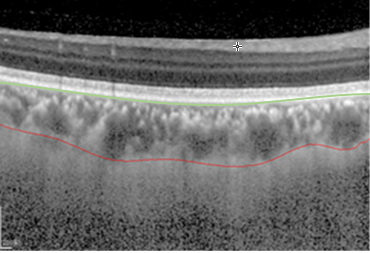 |
| 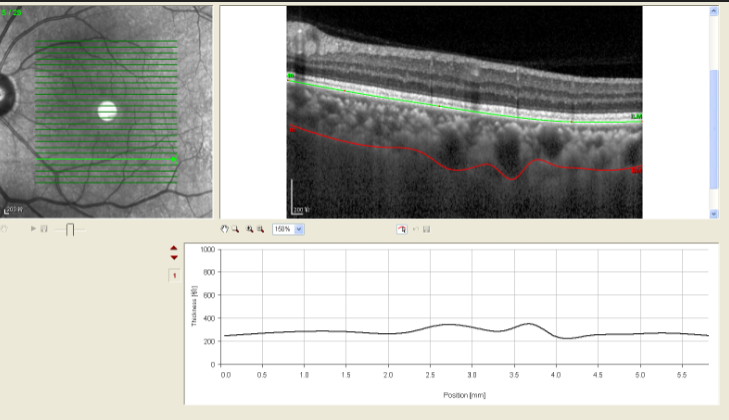 | 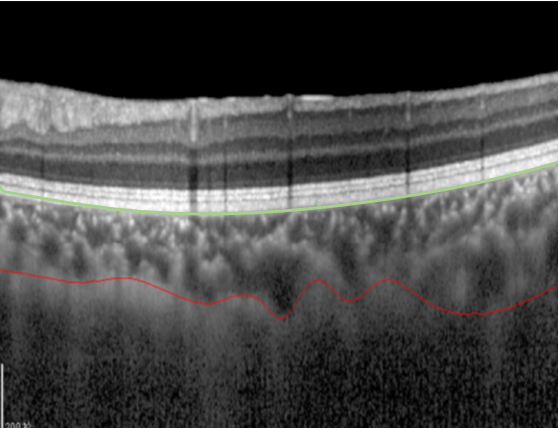 |
| **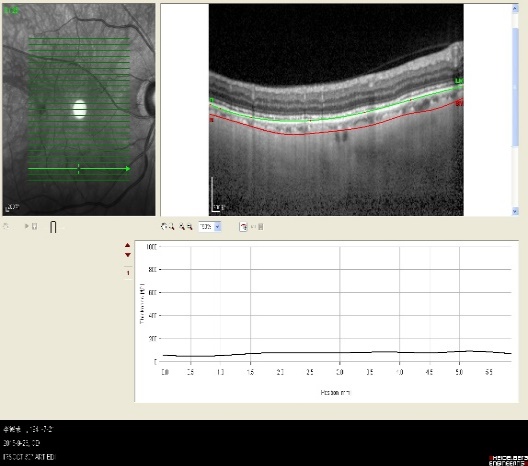** | 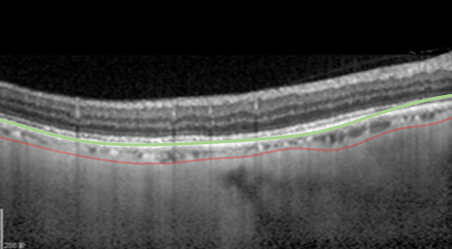 |
| **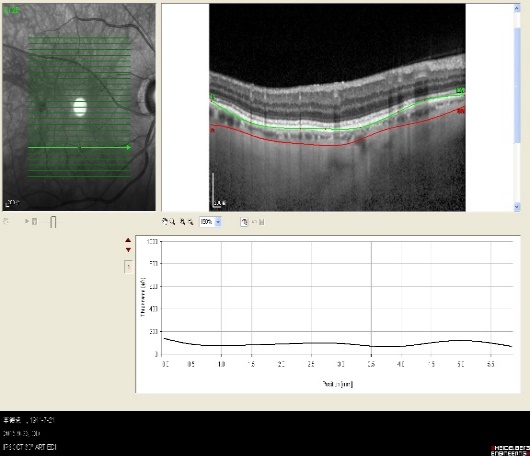** | 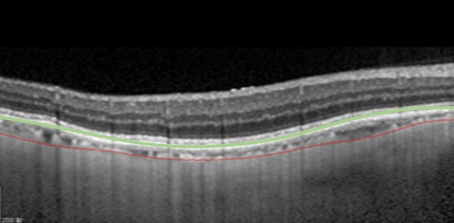 |
| **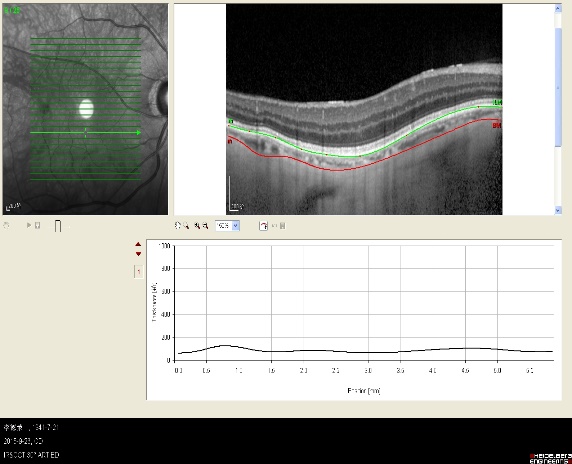** | 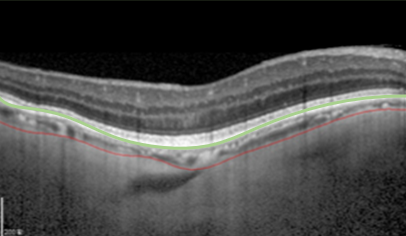 |
| **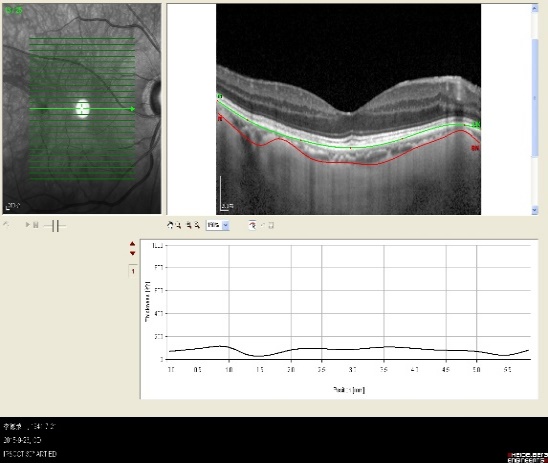** | 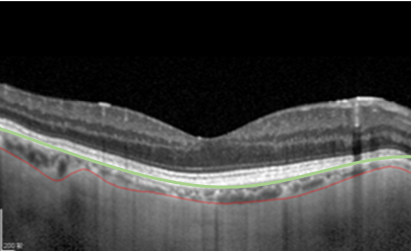 |
| **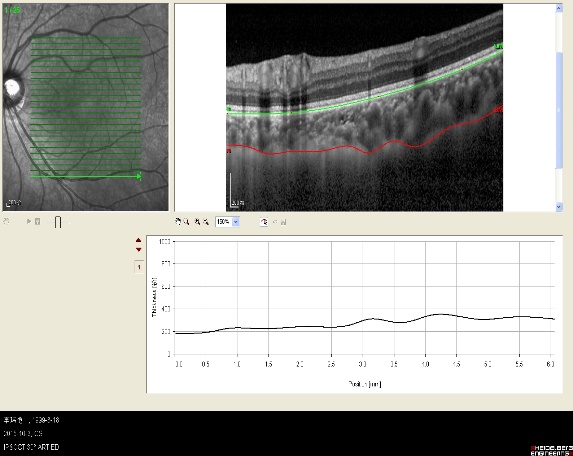** | 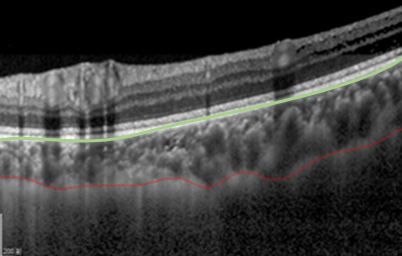 |
| **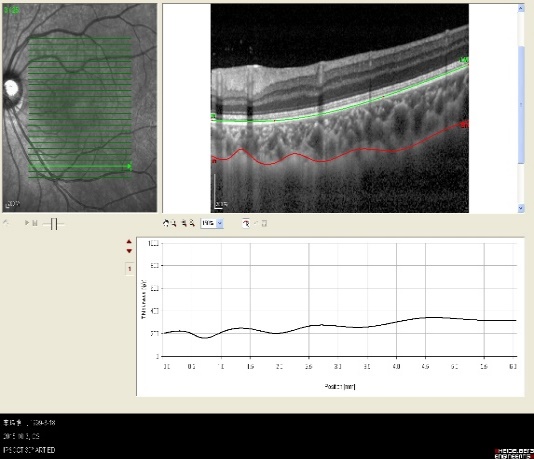** | 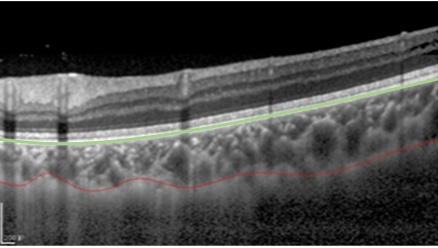 |
| **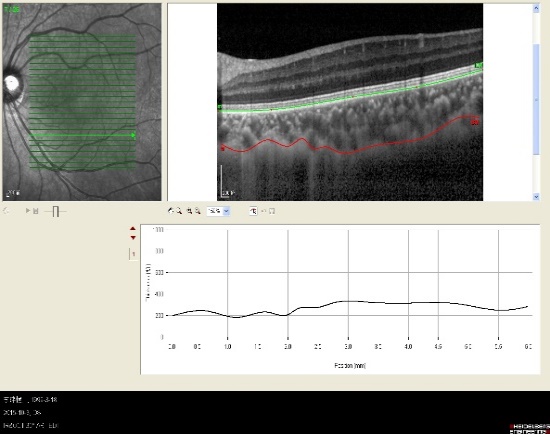** | 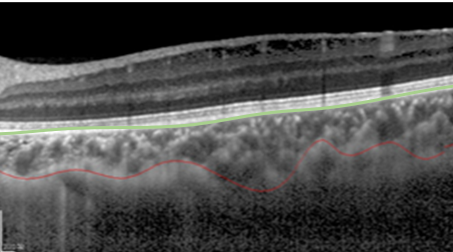 |
| **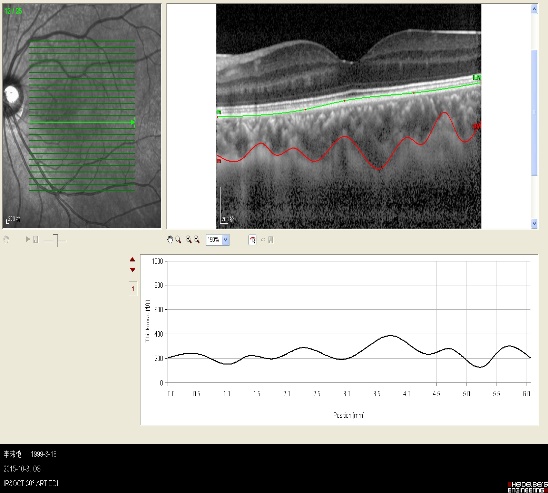** | 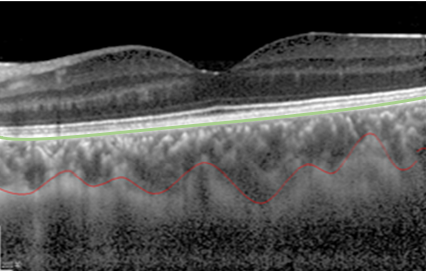 |

**Legend Description:** The supplementary results contain two columns, the first column contains the manual segmentation performed by the ophthalmologists whereas the second column represents the segmentation results acquired through the proposed method. The manual segmentation shows two annotated boundaries, one is in green color that represents the Bruch’s Membrane and another in red color representing the choroid. In the manual segmentation the x-axis of the graph represents the position of the layers whereas y-axis represents the thickness. Same color scheme is used in the results achieved from the proposed method, green represents Bruch’s Membrane Boundary whereas choroid is marked in red color. Based on the comparison between the manual segmentation and results acquired from the proposed method, it can be observed that the proposed method performs quite similar as the manual segmentation.
